# The Mycobacterial LysR-Type Regulator OxyS Responds to Oxidative Stress and Negatively Regulates Expression of the Catalase-Peroxidase Gene

**DOI:** 10.1371/journal.pone.0030186

**Published:** 2012-01-17

**Authors:** Yuqing Li, Zheng-Guo He

**Affiliations:** National Key Laboratory of Agricultural Microbiology, Center for Proteomics Research, College of Life Science and Technology, Huazhong Agricultural University, Wuhan, Hubei, China; Vrije Universiteit Brussel, Belgium

## Abstract

Protection against oxidative stress is one of the primary defense mechanisms contributing to the survival of *Mycobacterium tuberculosis* in the host. In this study, we provide evidence that OxyS, a LysR-type transcriptional regulator functions as an oxidative stress response regulator in mycobacteria. Overexpression of OxyS lowers expression of the catalase-peroxidase (KatG) gene in *M. smegmatis*. OxyS binds directly with the *katG* promoter region and a conserved, GC-rich T-N_11_-A motif for OxyS binding was successfully characterized in the core binding site. Interestingly, the DNA-binding activity of OxyS was inhibited by H_2_O_2_, but not by dithiothreitol. Cys25, which is situated at the DNA-binding domain of OxyS, was found to have a regulatory role for the DNA-binding ability of OxyS in response to oxidative stress. In contrast, the other three cysteine residues in OxyS do not appear to have this function. Furthermore, the mycobacterial strain over-expressing OxyS had a higher sensitivity to H_2_O_2_.Thus, OxyS responds to oxidative stress through a unique cysteine residue situated in its DNA-binding domain and negatively regulates expression of the *katG* gene. These findings uncover a specific regulatory mechanism for mycobacterial adaptation to oxidative stress.

## Introduction

*Mycobacterium tuberculosis*, the causative microbe of tuberculosis (TB), has a unique ability to survive and persist within host cell macrophages for long periods of time [1]. It can evade the host immune system by preventing phagosomal maturation and resist killing by reactive oxygen [2] and nitrogen intermediates [3]. Protection against oxidative stress is one of the primary defense mechanisms contributing to the survival of *M. tuberculosis* in the host [4].

In several bacteria, KatG, which encodes a catalase-peroxidase, is important for protection against oxidative stress. *katG* was shown to have a role in protecting the bacterium against micromolar concentrations of H_2_O_2_ in *Xanthomonas campestris* pv. Campestris [5]. A knockout mutation in *katG* that causes loss of catalase-peroxidase activity correlates with increased susceptibility to H_2_O_2_. *katG* expression is induced by oxidants in an OxyR-dependent manner [5]. It is also required for the activation of INH (isoniazid, a first-line anti-tuberculosis drug) in *M. tuberculosis* [6]. Expression of *katG* has been found to be negatively regulated by FurA in both *M. tuberculosis* and *M. smegmatis* [7]–[11]. FurA is a homologue of the ferric uptake regulator Fur and is encoded by a gene located immediately upstream of *katG*. The regulation of *katG* expression by FurA has been shown to be induced upon oxidative stress under the control of *pfurA*, located immediately upstream of the *furA* gene, but not of *pkatG*, located within the terminal region of the *furA* coding sequence [8]. Master et al. proposed that a *katG* promoter was present in this region [12]. It was also argued that the 5′ end of *katG* mRNA is generated by processing instead of transcription initiation [10]. Recently, a transcript analysis of the *furA-katG* loci revealed that *katG* is transcribed independent of *furA* in a fast-growing *Mycobacterium* sp. strain JC1 DSM 3803 [13]. However, the mechanism and the transcription factor for *furA*-independent regulation of *katG* remain to be characterized in mycobacteria [7], [12].

In several bacteria the expression of *katG* or *ahpC* is regulated in a FurA-independent manner by OxyR [14], [15], a well-characterized regulator responsible for adaptation to oxidative stress [16]. However, both *M. tuberculosis* and *M. smegmatis* lack a functional OxyR [11], [15], [17]. LysR-type transcriptional regulators (LTTRs) are thought to constitute the largest family of transcriptional regulators, and their functional orthologs have been identified in diverse bacterial genera, archaea and eukaryotic organisms. They regulate diverse functions including oxidative stress response, cell division, virulence, and secretion, among others [18]. Domenech *et al.* showed that OxyS, a *M. tuberculosis* LysR-type regulator, contains OxyR-like DNA binding domain and plays a role in response to oxidative stress in *M. tuberculosis* [17]. Overproduction or depletion of OxyS in *M. tuberculosis* did not affect susceptibility to isoniazid but increasing the concentration of OxyS lowered levels of the alkyl hydroperoxide reductase, AhpC, and rendered the tubercle bacillus more susceptible to organic hydroperoxides [17]. The introduction of *oxyS* on a multicopy plasmid did not affect catalase-peroxidase activity or KatG levels in the absence of peroxide. In the presence of peroxide they saw an increase in KatG protein level. They deleted the *oxyS* gene from the chromosome of *M. tuberculosis* and found no change in catalase-peroxidase activity, concluding that *oxyS* was not the main regulator of *katG*, but could be involved in a coregulatory process [17]. However, they mentioned that OxyS could not be purified successfully in *E. coli* and its biochemical characteristics remained unclear.

Here, we report that we have successfully purified soluble OxyS and its mutant forms from an *E. coli* expression system and characterized it as an oxidative stress response regulator in *M. tuberculosis*. OxyS has been shown to bind directly with the *katG* promoter region and the conserved binding site for OxyS in the promoter region of *katG* was successfully mapped out. A regulatory cysteine in the DNA-binding domain of OxyS was found to be important for its response to oxidative stress. Furthermore, we examined the effect of the expression level of OxyS on the mycobacterial sensitivity to H_2_O_2_. Our findings suggest that OxyS is a negative regulator of *katG* in mycobacteria.

## Results

### OxyS is a conserved mycobacterial LysR-type regulator and is involved in regulation of *katG*

**Figure 1:**
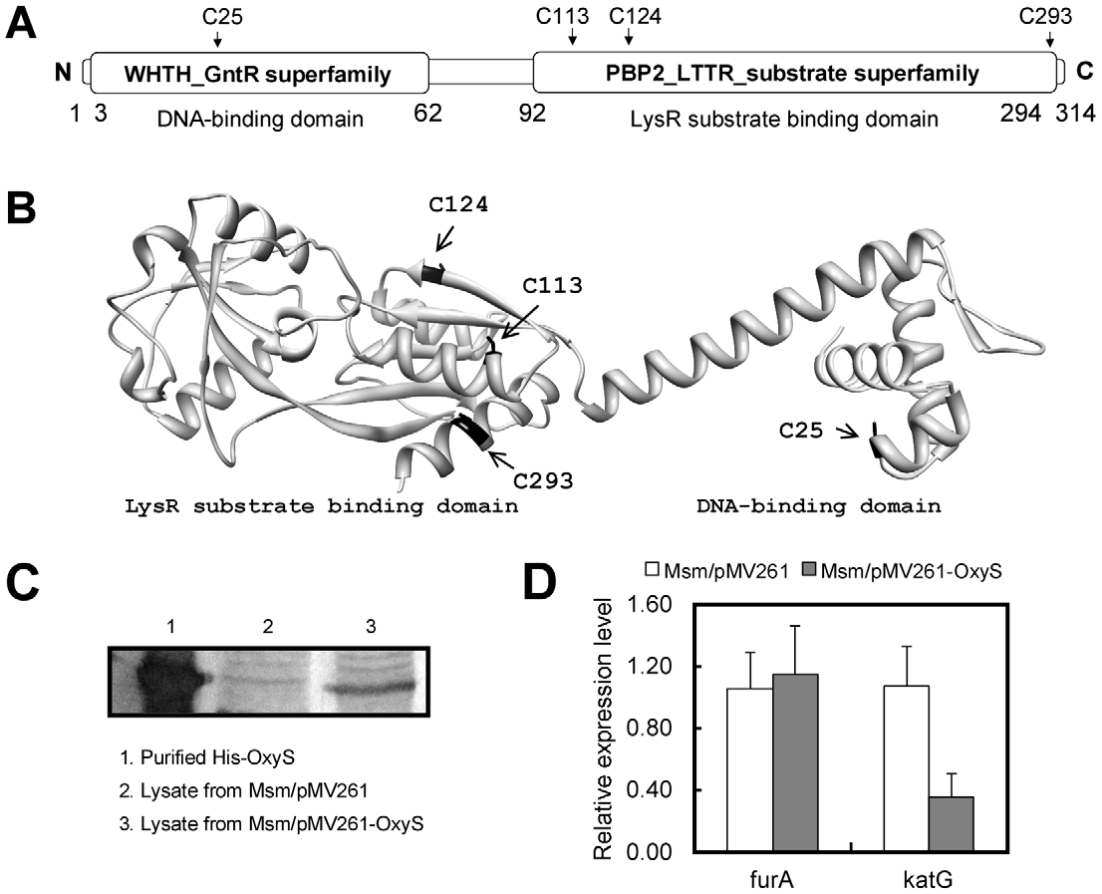
OxyS is a LysR-type regulator in *M. tuberculosis* and is involved in regulation of *katG*. (**A**) Conserved domain analysis of *M. tuberculosis* OxyS. The conserved domains in the N-terminus and C-terminus of OxyS were found by searching the CDD database on the NCBI website. (**B**) The modeled structure of OxyS was obtained using the automated comparative protein modeling web server SWISS-MODEL [34] and CbnR protein (a LysR family transcriptional regulator in Ralstonia eutropha NH9) [35] as a template (PDB ID: 1iz1). (**C**) Detection of OxyS protein by western blotting in the recombinant mycobacterial strains. An inducible system for conditional gene over-expression in mycobacteria [36] was used to over-express OxyS in *M. smegmatis*. Lane 1, purified His-OxyS; Lane 2, cell lysate from Msm/pMV261; Lane 3, cell lysate from Msm/pMV261-OxyS. (**D**) qRT-PCR assays conducted to analyze changes in gene expression in *M. smegmatis*. The experiment was carried out as described in the “Materials and Methods” section. The 16S rRNA gene, *rrs*, was used as an internal control for normalization. Target genes were amplified using specific primers. Expression levels of all genes were normalized to the levels of 16S rRNA gene transcripts, and fold-changes in expression of each gene were calculated. Representative data are shown.

Using a previously developed bacterial one-hybrid system, we have successfully isolated a number of novel transcription factors involved in the regulation of virulence genes in *M. tuberculosis* [19]. Interestingly, among the many newly identified transcription factors, we found OxyS was involved in the regulation of *katG*. The *M. tuberculosis* OxyS protein shares strong sequence identity with the LysR-type transcriptional regulator family (conserved domains database (CDD), NCBI). As shown in Fig. 1A and 1B, the N-terminal part of OxyS contains the helix-turn-helix motif and the C-terminal region contains a LysR substrate-binding domain which is structurally homologous to type-2 periplasmic binding proteins. Furthermore, the N-terminal helix-turn-helix motif of OxyS was found to be highly conserved among several different mycobacterial species (Fig. S1, Table 1).

**Table 1:** List of identified OxyS orthologous proteins from different mycobacteria species.

NCBI Accession Number	Name	Identity	Length	Mycobacteria species
NP_214631	OxyS (Rv0117)	100%	314 aa	*Mycobacterium tuberculosis* H37Rv
NP_853788	OxyS (Mb0121)	100%	314 aa	*Mycobacterium bovis* AF2122/97
YP_908187	OxyS (MUL_4805)	85%	252 aa	*Mycobacterium ulcerans* Agy99
ZP_04746764	OxyS (MkanA1_010100002242)	82%	310 aa	*Mycobacterium kansasii* ATCC 12478
YP_001848639	OxyS (MMAR_0317)	82%	405 aa	*Mycobacterium marinum* M
NP_302698	——	82%	310 aa	*Mycobacterium leprae* TN
ZP_05227802	OxyS_1 (MintA_010100022919)	80%	309 aa	*Mycobacterium intracellulare* ATCC 13950
NP_962456	OxyS_1 (MAP3522)	76%	312 aa	*Mycobacterium avium* subsp. paratuberculosis K-10
YP_884572	MSMEG_0156	62%	308 aa	*Mycobacterium smegmatis* str. MC^2^ 155

A previous study implied that OxyS is involved in regulation of KatG activity in *M. tuberculosis* [17]. Since the regions upstream of the *katG* gene in *M. tuberculosis* and *M. smegmatis* are highly conserved [8] (Fig. S2) we decided to explore that further by over-expressing *M. tuberculosis* OxyS in *M. smegmatis* to determine the physiological role of OxyS. As shown in Fig. 1C, OxyS reached a high level of expression in the recombinant strain as revealed by Western blotting assays using anti-OxyS serum. Notably, qRT-PCR (Quantitative real-time PCR) analysis indicated that the level of *katG* expression in the recombinant *M. smegmatis* strain was only 36% of that of the control strain (Fig. 1D). In contrast, the expression level of *furA* showed no significant change when compared with *M. smegmatis* strains harboring empty vectors. These results indicate that the expression of *katG* is negatively regulated by OxyS, while the *furA* expression is not regulated by OxyS.

### OxyS directly targets the promoter region of *katG* in *M. tuberculosis* and *M. smegmatis*

**Figure 2:**
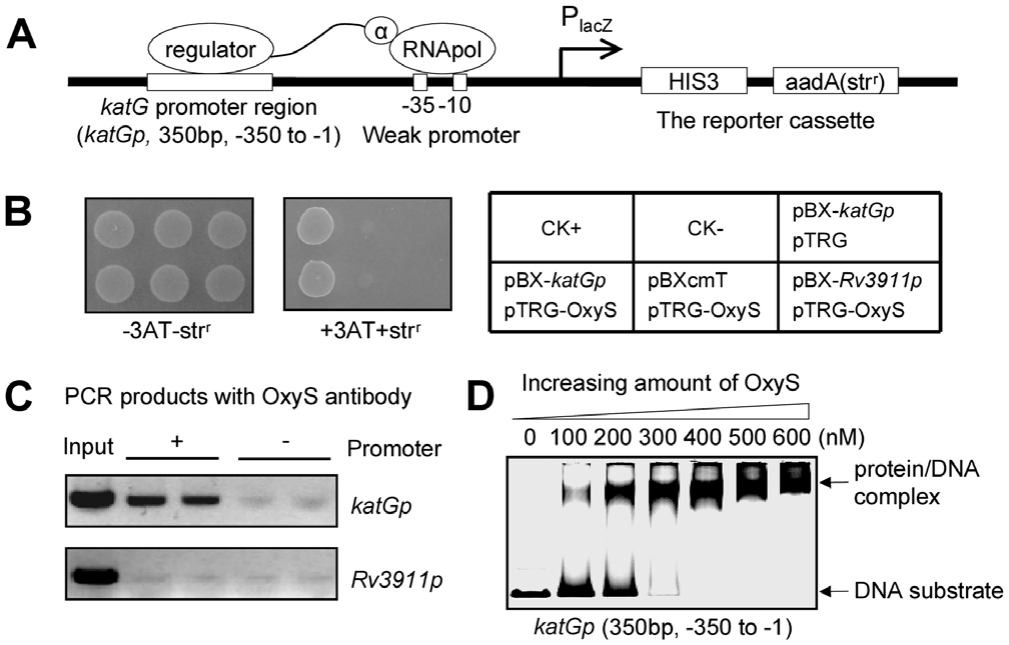
OxyS interacts with the regulatory region of *katG* in *M. tuberculosis*. (**A**) The regulatory sequence of the *katG* gene was cloned into the upstream of *HIS3-aadA* reporter genes of the bacterial one-hybrid reporter vector pBXcmT [19]. (**B**) The interaction between OxyS and the promter region of *katG* was measured by bacterial one-hybrid analysis [19]. Left panel: bacterial one-hybrid plates. Right panel: an outline of the plates in the left panel. Each unit represents the corresponding co-transformant in the plates. CK+: co-transformant containing pBX-Rv2031p and pTRG-Rv3133c as a positive control. CK−: co-transformant containing pBX-Rv2031p and pTRG-Rv3133c-deltaC as a negative control [19]. *Rv3911p* (the promoter of the *Rv3911* gene) was also used as a negative control. (**C**) *in vivo* ChIP assays for the interaction of OxyS with the *katG* promoter in *M. tuberculosis*. DNA recovered from the immunoprecipitates was amplified with primers specific for either *katGp* or a negative control promoter *Rv3911p*. ‘+’ refers to the immunoprecipitate obtained with OxyS antibodies, whereas ‘−’ refers to the control in which ChIP was carried out without any primary antibodies. ‘Input’ refers to total genomic DNA prior to IP reaction and was used as a positive control in PCR. (**D**) EMSA assays for the binding of OxyS to the *katG* promoter. The EMSA reactions (10 µl) for measuring mobility shift contained FITC-labeled DNA and increasing amount of OxyS (100 nM, 200 nM, 300 nM, 400 nM, 500 nM and 600 nM). The protein/DNA complexes are indicated by arrows on the right of the panels.

To examine whether a direct interaction occurs between OxyS and the regulatory sequence of the *M. tuberculosis katG* gene, a bacterial one-hybrid assay was conducted by cloning the *katG* promoter region upstream of *HIS3-aadA* reporter genes of the bacterial one-hybrid reporter vector pBXcmT (Fig. 2A) [19]. As shown in Fig. 2B, co-transformant strains with the *katG* promoter and OxyS grew well on selective plates. In contrast, the strains containing either OxyS alone or the *katG* promoter alone did not grow on selective plates. In addition, co-transformant strains containing the promoter of *Rv3911* (*Rv3911p*), an unrelated promoter, and OxyS did not grow either (Fig. 2B). These results indicate that OxyS can specifically interact with the promoter of the *katG* gene.

Using anti-OxyS antibodies and ChIP (chromatin immunoprecipitation) assays, we characterized the association of OxyS with the *katG* promoter *in vivo* in *M. tuberculosis*. Fig. 2C (top panel) shows that the signal for *katG* promoter was enriched in the anti-OxyS immunoprecipitate compared with that in the control sample without any antibody. In contrast, no obvious signal was observed for the promoter of *Rv3911* (*Rv3911p*) in both the anti-OxyS and the control immunoprecipitates (Fig. 2C, bottom panel). We successfully purified soluble His-tagged OxyS from an *E. coli* expression system by inducing the expression of MtbOxyS protein under low temperature conditions (Fig. S3). Binding of the purified OxyS protein with the *katG* promoter was then confirmed by further EMSA (electrophoretic mobility shift assay) experiments. As shown in Fig. 2D, when increasing amounts of OxyS (100–600 nM) were added into the reactions, shifted bands corresponding to the OxyS/*katG* promoter complex were observed and a corresponding increase in the percentage of protein/DNA complexes was seen. Using competitive EMSA assays (Fig. S4C), we comfirmed that this interaction was specific. Notably, the interaction of OxyS with the promoter of Msm*katG* was also confirmed in the recombinant OxyS-overexpressing *M. smegmatis* strain by both ChIP and EMSA assays (Fig. S5).

Taken together, these results indicate that OxyS can specifically interact with the promoter of the *katG* gene *in vivo* and *in vitro*, and this direct interaction is conserved in both *M. tuberculosis* and *M. smegmatis*.

### Characterization of the binding sites and sequence motifs for OxyS in the promoter region of *katG*

To identify the binding sites for OxyS in the promoter region of *katG* (*katGp*), we first obtained two short DNA fragments (*katGp1* and *katGp2*) of the *katG* promoter (Fig. S4A). As shown in Fig. S4B, OxyS could form specific protein/DNA complexes with the substrates *katGp* and *katGp2*. In contrast, no binding activity was observed for the substrate *katGp1*. In addition, unlabeled *katGp* and *katGp2* could competitively inhibit the binding of OxyS with the labeled DNA substrate, while unlabeled *katGp1* had no effect on OxyS binding (Fig. S4C). This indicates that the −1 to −180 region (*katGp2*) of the *katG* promoter contains the binding site for OxyS.

**Figure 3:**
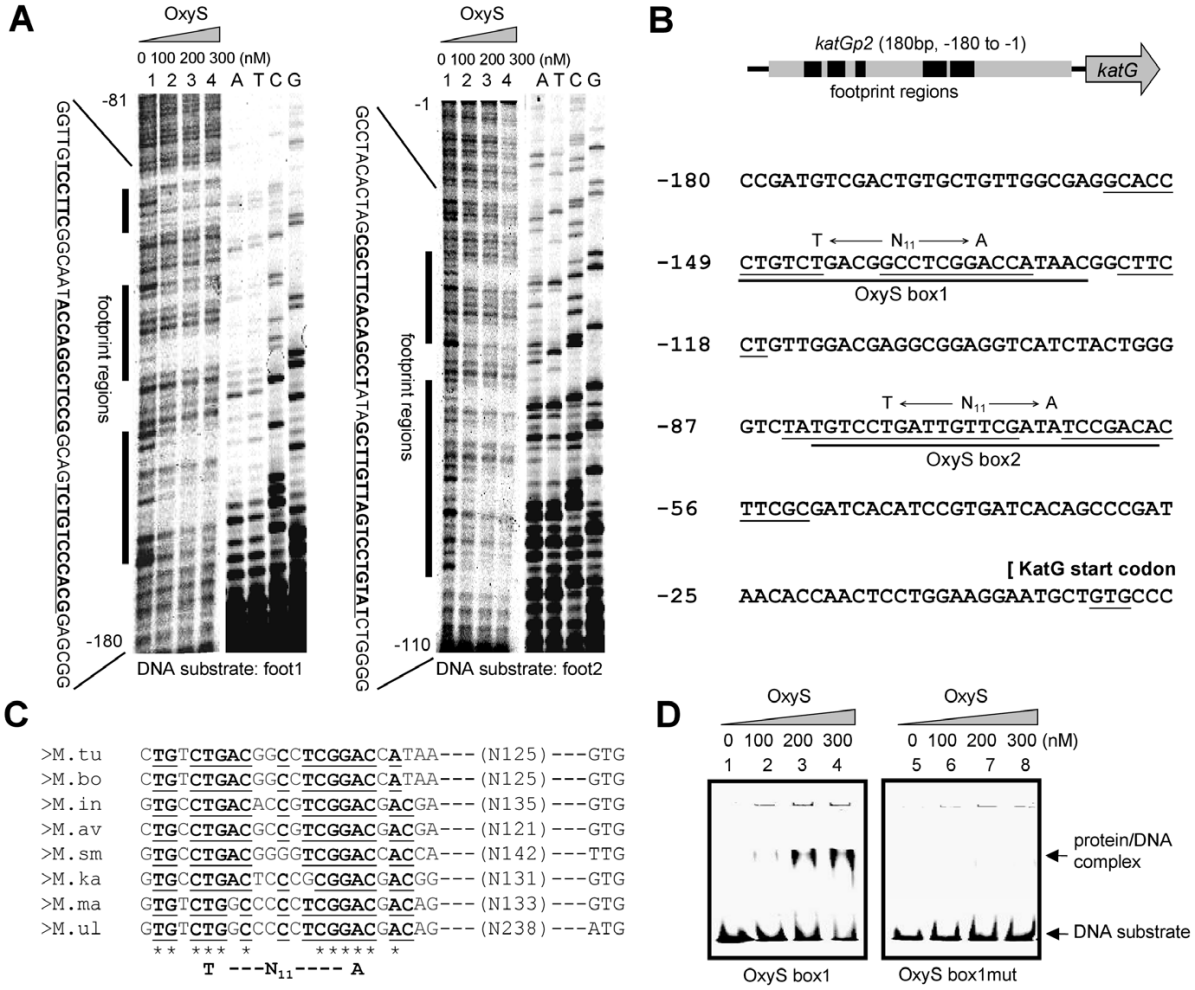
Identification of the binding sites for OxyS in the promoter region of *katG*. (**A**) DNase I footprinting assays were employed to assess the binding sequence of OxyS. The experiments were carried out as described under the “Materials and Methods” section. The ladders are shown in the right panel and the nucleotide sequences obtained are listed. The protected regions are underlined. (**B**) Summary of OxyS footprinting analysis in the *M. tuberculosis katG* promoter region. The DNA sequence where OxyS was found to bind corresponds with the *katG* promoter region from −180 to −1. OxyS footprint regions (underlined) and the position of the *katG* translation start site are indicated. The T-N_11_-A motif conserved in the DNA binding sites of LysR regulators is indicated on the sequence. Two OxyS binding boxes are indicated. (**C**) A blast assay for the conserved sequence motif recognition by OxyS (OxyS box1) among different mycobacterial species. Sequence alignment was carried and visualized locally using the BioEdit software. The conserved T-N_11_-A DNA-binding site in LysR regulators is shown at the bottom. M. tu, *Mycobacterium tuberculosis* H37Rv; M. bo, *Mycobacterium bovis* AF2122/97; M. in, *Mycobacterium intracellulare* ATCC 13950; M. av, *Mycobacterium avium* subsp. paratuberculosis K-10; M. sm, *Mycobacterium smegmatis* str. mc^2^155; M. ka, *Mycobacterium kansasii* ATCC 12478; M. ma, *Mycobacterium marinum* M; M. ul, *Mycobacterium ulcerans* Agy99. (**D**) DNA-binding assays for OxyS on *OxyS box1* and *OxyS box1mut* substrates. The EMSA reactions (10 µl) for measuring mobility shift contained FITC-labeled DNA substrate and increasing amount of OxyS (0 nM, 100 nM, 200 nM and 300 nM). The conserved “TG” and “GA” in *OxyS box1* were replaced by “CC” in *OxyS box1mut* substrate. The protein/DNA complexes are indicated by arrows on the right of the panels.

Two fragments—foot1 and foot2—were further designed (Fig. S4A) to precisely determine the binding sites for OxyS in the *katG* promoter by DNase I footprinting assays. As shown in Fig. 3A, the foot1 and foot2 fragments were incubated with increasing amounts of the OxyS protein and digested with DNase I. Several regions were protected from digestion in the presence of the OxyS protein (Fig. 3A). Two OxyS binding boxes, named OxyS box1 and OxyS box2, were further found in the protected regions, as indicated in Fig. 3B. The core OxyS binding sites of *M. tuberculosis katG* also contained the LysR-binding motif T-N_11_-A, which has previously been shown to be a classic LTTR box [18].

A blast assay for the binding sequence for OxyS (OxyS box1) among different mycobacterial species revealed a conserved, GC-rich T-N_11_-A motif (G[C]T**GC[T]CTG**A[G]CNC[G]C [G]C[G]T[G]**CGGAC**G[C]AC[T]A[G]A[G]) (Fig. 3C). Specific binding of OxyS to the motif was then confirmed by base replacement and EMSA assays. As shown in Fig. 3D, when the conserved “TG” and “GA” in OxyS box1 were replaced by “CC”, the OxyS protein lost its ability to bind to the mutant DNA substrate as evidenced by the result of our EMSA assay that specific protein/DNA complexes were observed for the wild type substrate (Fig. 3D, left panel), but no such complex was observed for the mutant substrate (Fig. 3D, right panel).

### The DNA-binding ability of OxyS is inhibited by H_2_O_2_ and Cys25 is a regulatory residue

**Figure 4:**
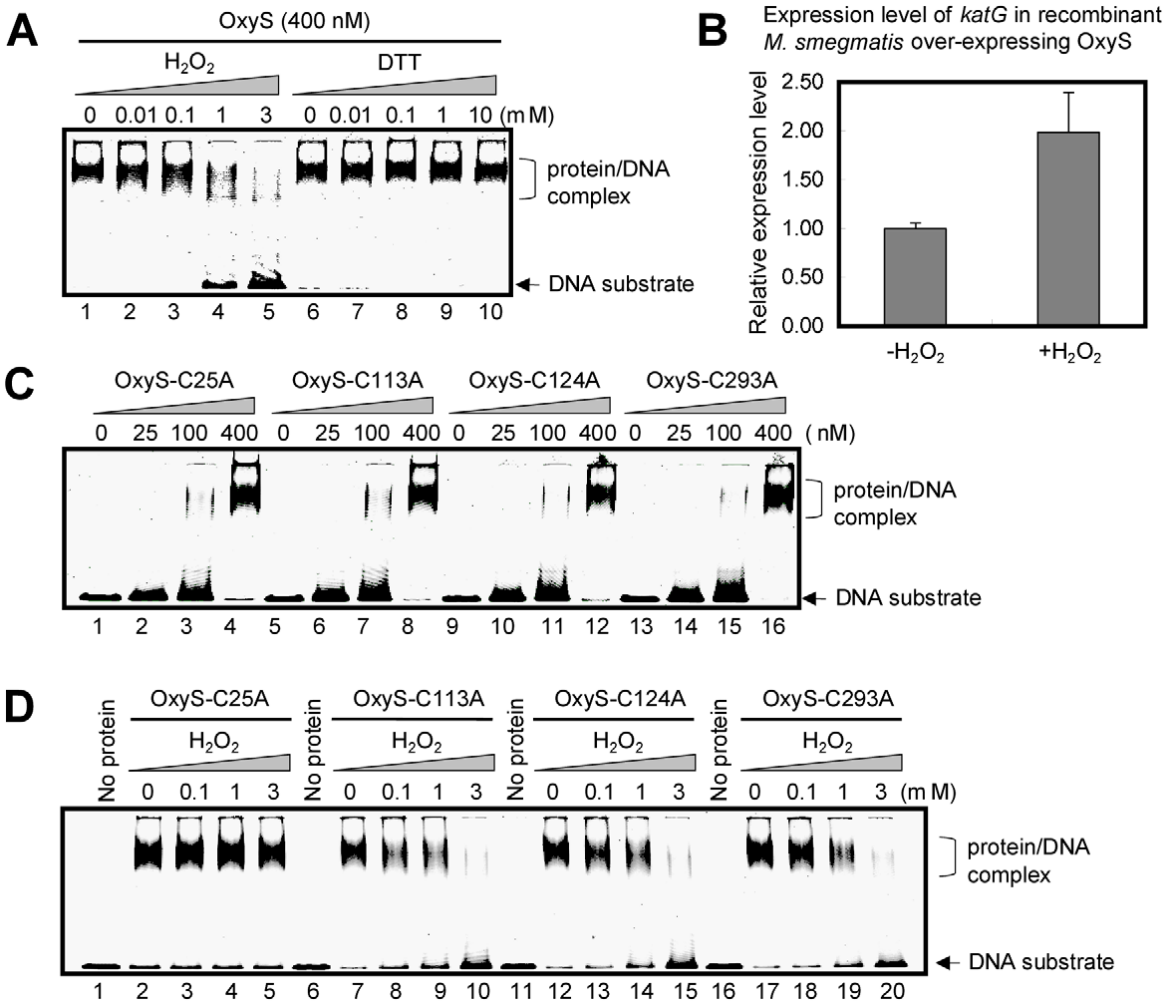
Effect of H_2_O_2_ on the DNA binding ability of OxyS and its mutant variants. (**A**) Effects of H_2_O_2_ and DTT on the DNA binding activity of OxyS were measured by EMSA. The concentration of OxyS in lanes 1 to 10 was 400 nM. The increasing concentration of H_2_O_2_ or DTT is indicated at the top of the panels. (**B**) qRT-PCR assays conducted to analyze changes in gene expression of *katG* in *M. smegmatis* over-expressing OxyS after H_2_O_2_ (2 mM) treatment. The experiment was carried out as described in the “Materials and Methods” section. Expression levels of all genes were normalized to the levels of 16S rRNA gene transcripts, and the fold-changes in expression were calculated. Representative data are shown. (**C**) EMSA assays for the interactions of the mutant proteins with *katGp*. The protein/DNA complexes are indicated by arrows on the right of the panels. The increasing concentrations of proteins are indicated at the top of the panels. (**D**) Effects of H_2_O_2_ on the DNA binding activity of the mutant proteins. The concentration of proteins was 400 nM. The concentration of H_2_O_2_ is indicated at the top of the panels. The protein/DNA complexes are indicated by arrows on the right of the panels.

We assayed the effect of redox reagents on the DNA-binding ability of OxyS. As shown in Fig. 4A, a stepwise decrease in amounts of the specific protein/DNA complex was observed as 0.01–3 mM H_2_O_2_ was added to the DNA-binding reaction mixture (Fig. 4A, lanes 1–5). In contrast, no effect was observed when DTT (dithiothreitol) was added to the reaction mixture (Fig. 4A, lanes 6–10), indicating that the ability of OxyS to bind DNA was specifically reduced by H_2_O_2_. We also examined the expression level of *katG* in the *M. smegmatis* strain under oxidative stress (due to OxyS over-expression) by qRT-PCR assays. As shown in Fig. 4B, the expression level of *katG* increased 2-fold after 2 mM H_2_O_2_ treatment, indicating that the negative regulation of *katG* by OxyS was eliminated.

The OxyS protein contains four cysteines (Fig. 1B), and two of them (Cys25 and Cys113) were found to be conserved among several mycobacterial OxyS orthologs (Fig. S1). Cysteine residues have been shown to be responsible for redox-sensing in many transcriptional regulators [20]–[22]. To identify the sites responsible for sensing oxidative stress in OxyS, site-directed mutations were introduced into these cysteine residues in the *oxyS* gene. The mutant proteins were expressed and successfully purified from an *E. coli* expression system (Fig. S3). As shown in Fig. 4C, all the mutant proteins maintained their DNA-binding ability when compared with wild-type protein. We examined the effect of these mutations on the ability of OxyS to bind to DNA in response to H_2_O_2_. Cys113, Cys124 and Cys293 mutations did not change the sensitivity of OxyS to H_2_O_2_ (Fig. 4D). Interestingly, the DNA-binding activity of the mutant protein OxyS-Cys25A, in which the mutation was situated in the DNA-binding domain of OxyS, was not affected by H_2_O_2_ (Fig. 4D) indicating that it lacked the ability to respond to the oxidative signal. These results indicate that the Cys25 residue is involved in the regulation of the DNA-binding ability of OxyS under oxidative stress, while the other three cysteine residues are dispensable.

The fate of OxyS and its mutant variants after peroxide treatment was further determined by native-PAGE assays. As shown in Fig. S6, addition of 3 mM H_2_O_2_ (lane 3) changed the electrophoretic mobility of OxyS and all its mutant variants. In contrast, addition of 3 mM DTT (lane 2) neither altered the electrophoretic pattern nor changed the electrophoretic mobility of MSMEG_6092, a DNA binding protein from *M. smegmatis* with no cysteine residues (Fig. S6). These results indicate that purified OxyS proteins are in a reduced state and are capable of redox-sensing.

Taken together, our results lend support to a model in which all the cysteine residues in OxyS can sense oxidative signal, but Cys25 is the only regulatory cysteine residue capable of both sensing the oxidative signal and regulating the ability of OxyS to bind to DNA under oxidative stress.

### OxyS-Cys25A lacks the ability to respond to the oxidative signal *in vivo* in *M. smegmatis*

**Figure 5:**
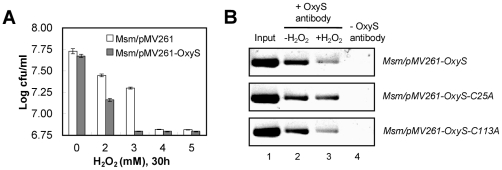
Effects of H_2_O_2_ on the growth of recombinant mycobacterial strains and the interaction of OxyS with *katG* promoter in *M. smegmatis*. (**A**) Effects of H_2_O_2_ on the growth of recombinant mycobacterial strains. The antimicrobial activity of H_2_O_2_ against *M. smegmatis* was determined using a modified bacterial growth time course assay described under “Materials and Methods”. *M. smegmatis* strains were grown in LB at 37°C overnight. This culture was then diluted (1∶100) in 5 ml of fresh LB broth containing the indicated concentration of H_2_O_2_, and the culture was again incubated at 37°C with shaking at 220 rpm for three days. Aliquots were taken at the indicated times, and the number of CFUs per ml (cfu/ml) was determined. Each analysis was performed in triplicate. Symbols are the average of three replicates, and error bars indicate the SDs (Standard Deviation) of three replicate samples. The recombinant mycobacterial strains are indicated. (**B**) ChIP assays for the effect of H_2_O_2_ on the interaction of OxyS and its mutant proteins with *katG* promoter in *M. smegmatis*. The experiments were carried out as described under “Materials and Methods”. Recombinant *M. smegmatis* strain over-expressing OxyS or its mutant proteins was treated with H_2_O_2_ (lane 2, 0 mM; lane 3, 2 mM) before cross-link. DNA recovered from the immunoprecipitates was amplified with primers specific for *MsmkatGp*. ‘+’ refers to the immunoprecipitate obtained with OxyS antibodies, whereas ‘−’ refers to the control in which ChIP was carried out without any primary antibodies. ‘Input’ refers to total genomic DNA prior to IP reaction and was used as a positive control in PCR.

We first examined the effect of over-expressing OxyS on oxidative stress response in *M. smegmatis* using a modified bacterial growth time course assay. As shown in Fig. 5A, we observed that the *M. smegmatis* strains over-expressing OxyS were more sensitive to H_2_O_2_ as 2–5 mM H_2_O_2_ was added to the medium if compared with the control strain. This is consistent with a previous observation [17]. The findings of our detailed bacterial growth time course assays with increasing concentrations of H_2_O_2_ also support the same conclusion (Fig. S7). As shown above, overexpression of OxyS lowered expression of the *katG* gene in *M. smegmatis* (Fig. 1D) and OxyS-Cys25 is a regulatory cysteine residue (Fig. 4D). Thus, we further compared the DNA-binding ability of mutant OxyS proteins with that of the wild type protein for the promoter of *katG in vivo* in response to H_2_O_2_. As shown in Fig. 5B, when over-expression of OxyS-WT or OxyS-C113A in *M. smegmatis*, the signal for *katG* promoter with 2 mM H_2_O_2_ treatment was reduced substantially in the anti-OxyS immunoprecipitate if compared with that in the control sample without H_2_O_2_ treatment. In contrast, no obvious signal change for the enriched *katG* promoter in response to H_2_O_2_ was observed for over-expression of OxyS-C25A (Fig. 5B). Therefore, the mutant protein OxyS-Cys25A lacked the ability to respond to the oxidative signal *in vivo* in *M. smegmatis*.

## Discussion

*M. tuberculosis* can resist the damaging effects of reactive oxygen and nitrogen intermediates produced in the host cells and thus survive successfully for long periods of time [2], [3]. However, the transcriptional regulatory processes involved in this mycobacterial defense mechanism are still unclear. In this study, we show that OxyS, a LysR-type transcriptional regulator, is an oxidative stress response regulator in *M. tuberculosis* that binds directly with the *katG* promoter region, which is located in the terminal part of the *furA* coding region. Furthermore, the DNA-binding activity of OxyS was found to be inhibited by H_2_O_2_. We characterized a conserved residue, which is situated within the DNA-binding domain of OxyS, important for DNA binding in response to oxidative stress. Finally, the expression level of *katG* was greatly reduced in the mycobacterial strain overexpressing OxyS and the recombinant strain had an elevated sensitivity to H_2_O_2_.Taken together, our results indicate that OxyS functions as a negative regulator of *katG* in response to oxidative stress in mycobacteria.

Response to oxidative stress plays an important role in pathogen-host interaction during infection [4] and in determining the intrinsic susceptibility of mycobacterial species to INH [6]. Both in *M. tuberculosis* and in *M. smegmatis*, the expression of *katG* is induced upon oxidative stress under the control of *pfurA*, and is negatively regulated by FurA [7], [8]. However, it has been suggested that *katG* can be transcribed independent of *furA* in a fast-growing *Mycobacterium* sp. strain [13]. It has recently been reported that in *Caulobacter crescentus*, the expression level of the *katG* gene is positively regulated by OxyR in a FurA-independent manner. OxyR was found to bind to a canonical OxyR binding site in the promoter region of *katG*, and this interaction was redox dependent, as only an oxidized form of the protein could bind to the *katG* promoter [14]. In the present study, we found that the DNA binding ability of OxyS was inhibited, not stimulated, by H_2_O_2_. Moreover, in *M. smegmatis*, the expression level of *katG* decreased when OxyS was over-expressed, while the expression level of *furA* showed no significant change, suggesting that the expression of *katG* is negatively regulated by OxyS, and the *furA* expression is not regulated by OxyS. Notably, the expression level of *katG* increased 2-fold in the OxyS-overexpressed mycobacterial strain after H_2_O_2_ treatment (Fig. 4B). This result is consistent with an inhibitory effect of H_2_O_2_ on the DNA-binding ability of OxyS that we observed. Interestingly, Domenech and his colleagues also observed an increase in KatG activity upon H_2_O_2_ treatment in *M. tuberculosis* over-expressing OxyS [17]. In the present study, the morphology of *M. smegmatis* cells was also examined using scanning electron microscopy (SEM) to further investigate the reason for the increased sensitivity of *M. smegmatis* strains over-expressing OxyS to H_2_O_2_. Over-expression of OxyS in *M. smegmatis* had no obvious effect on cell morphology when compared with *M. smegmatis* cells harboring empty vectors (Fig. S8). Therefore, the effect of over-expression of OxyS on the sensitivity of *M. smegmatis* to H_2_O_2_ was not due to the changes in cell morphology.

In the current study, by using a bacterial one hybrid system, we found that the reporter genes (*HIS3-aadA*) downstream the *katG* promoter could be successfully activated (Fig. 2B), which indicating the existance of regulatory elements in this region. We also found using *in vivo* and *in vitro* assays that MtbOxyS can directly interact with the promoter region of *katG* by binding to a typical LysR type T-N_11_-A motif in the *katG* promoter. Two OxyS binding boxes in the promoter region of *katG* were identified. However, only one major protein/DNA complex band was observed in our EMSA assays (Fig. 2D). This may due to the different binding preference of OxyS to these boxes or the cooperative binding of these two boxes by OxyS. Indeed, through a blast assay of the *katG* promoters from different mycobacterial species, we found that OxyS box1 was the most conserved motif (Fig. 3C). This suggests a common regulatory mechanism of *katG* expression by OxyS among different mycobacterial species. Interestingly, the binding motif for OxyS identified in the current study also shows similarity to the binding motif for OxyR in *C. crescentus* [14].

Cysteine residues have been shown to be responsible for redox sensing [20]–[22]. OxyR senses H_2_O_2_ and is activated through the formation of a transient disulphide bond between Cys199 and Cys208 in its regulatory domain [23]. Chen *et al.* have recently reported an OxyR sensor with one cysteine residue, and divided the OxyR family into two classes: typical 2-Cys OxyR and 1-Cys OxyR [20]. In the present study, we found that all the cysteine residues in OxyS could sense the oxidative signals, but Cys25 was the only regulatory cysteine residue which could both sense the oxidative signal and regulate the DNA binding ability of OxyS under oxidative stress. This represents an interesting redox sensing and regulatory mechanism through a cysteine residue in the DNA binding domain in mycobacteria. The electrophoretic mobility of OxyS and its mutant variants were changed under oxidative stress and the DNA binding ability of OxyS was inhibited after H_2_O_2_ treatment. These characteristics are similar to that of FurA, an oxidative stress-responsive protein which regulates the expression of *katG* in *M. tuberculosis* [9]. The regions upstream of the *katG* gene in *M. tuberculosis* and *M. smegmatis* were found to be highly conserved [12], suggesting a putative common regulatory mechanism in these two species. In the present study, we found that OxyS could interact with the promoter of *katG* in both *M. tuberculosis* and *M. smegmatis*. Furthermore, we identified a LysR family transcriptional regulator (MSMEG_0156) which shares 62% amino acid identity with MtbOxyS in a blast assay (Table 1). The regulatory cysteine residue Cys25 is conserved in both of these proteins.

OxyR regulates KatG and other proteins induced by exposure to H_2_O_2_ in *E. coli* [24]. However, the *oxyR* gene in *M. tuberculosis* harbors multiple mutations [25], [26] that render it inactive. The alterations in *oxyR* are conserved in all members of the *M. tuberculosis* complex, including *M. africanum*, *M. bovis*, and *M. microti* [25], with minor variations [27]. Moreover, no potential OxyR-encoding sequences could be detected in *M. smegmatis* [28]. In contrast, intact *oxyR* genes have been found in *M. leprae* [25], [26] and *M. avium* [26]. In the present study, we showed that the OxyS proteins from both *M. tuberculosis* and *M. smegmatis* can respond to H_2_O_2_ stress and regulate the expression of the *katG* gene. This lends support to the interesting possibility that given the lack of functional OxyR, OxyS could be an alternative regulator of the response to oxidative stress in these mycobacterial species.

In conclusion, we have provided evidence to show that OxyS, a LysR-type transcriptional regulator, functions as an oxidative stress response regulator in *M. tuberculosis*. OxyS negatively regulates the expression of the *katG* gene and responds to oxidative stress through its unique cysteine residue situated in the DNA-binding domain. The DNA binding ability of OxyS was shown to be inhibited by H_2_O_2_. A conserved T-N_11_-A and GC-rich motif for OxyS binding in the promoter region of *katG* was characterized. The mycobacterial strain over-expressing OxyS had increased sensitivity to H_2_O_2_. These findings suggested a new function for the mycobacterial OxyS gene and a regulatory mechanism for their adaptation to oxidative stress.

## Materials and Methods

### Bacterial strains, plasmids, enzymes and chemicals

*Escherichia coli* BL21 (DE3) strains, purchased from Novagen, were used as the host strain to express *M. tuberculosis* OxyS. pBT, pTRG vectors and *E. coli* host strains XR were purchased from Stratagene. pET28a was purchased from Novagen. Restriction enzymes, T4 ligase, and modification enzymes were from TaKaRa Biotech. Pyrobest DNA polymerase and deoxynucleoside triphosphates (dNTPs) were purchased from TaKaRa Biotech. DNA purification kits were purchased from Watson Biotechnologies. All antibiotics were purchased from TaKaRa Biotech. Ni-NTA (Ni^2+^-nitrilotriacetate) agarose columns were obtained from Qiagen. All DNA and oligonucleotides were synthesized by Invitrogen (Table S1).

### Site-directed mutagenesis

To identify the oxidative stress-sensing sites, site-directed mutations were introduced into the cysteine residues in the *oxyS* gene by overlap PCR [29]. Sequences of mutated oligonucleotides are listed in Table S1. All fragments were ligated into pET28a and were subsequently sequenced to confirm the presence of the site-directed mutations.

### Cloning, expression and purification of recombinant proteins

The *M. tuberculosis oxyS* gene was amplified from genomic DNA using the High Fidelity PCR system (TaKaRa) with appropriate primers and cloned into the pET28a over-expression vectors to produce recombinant vectors (Table S2). *E. coli* BL21 (DE3) cells transformed with the recombinant plasmid were grown at 37°C for 5 hours in 1 liter of LB medium containing 30 µg/ml^−1^ kanamycin, and at 20°C for 15 hours after 0.5 mM IPTG (isopropyl β-D-1-thiogalactopyranoside) was added. Purification of recombinant proteins was carried out as described in a previous study [30]. Purified proteins were more than 99% pure as determined by SDS-PAGE. The gels were stained with coomassie blue R-250 and destined with 5% methanol, 10% acetic acid until the background was clear. Protein concentrations were determined by spectrophotometric absorbance at 260 nm according to Gill and Hippel [31]. Native PAGE for OxyS and its mutant proteins were performed at room temperature with the use of 12% non-denaturing polyacrylamide gels. Electrophoresis gels were run at a constant voltage of 125 V in 37 mM Tris–glycine buffer (pH 8.9) until the tracking dye reached the bottom of the gel. The gels were then stained and destained as described above.

### Bacterial one-hybrid analysis

The regulatory sequences of the *katG* gene or *Rv3911*, a sequence used as negative control, were cloned into the reporter genes upstream of *HIS3-aadA* of the reporter vector pBXcmT (Table S2) [19]. OxyS was fused to the N-terminal domain of the α-subunit of RNA polymerase in the pTRG vector (Stratagene). The bacterial one-hybrid assay was carried out as described in a previous study [19]. The reporter strain was co-transformed with pBX and pTRG recombinant plasmids. The colonies (reporter strain contains the indicated pBX and pTRG recombinant plasmids) were selected on plates containing 15 mM 3-AT (3-amino-1,2,4-triazole) and 8 µg/ml streptomycin, while plates that did not contain 3-AT and streptomycin were used as controls. Detection of protein-DNA interactions was based on transcriptional activation of the reporter genes, *HIS3* and *aadA*, which allows growth in the presence of 3-AT and streptomycin, respectively. Co-transformants containing pBX-Rv2031p and pTRG-Rv3133c were used as positive controls and were expected to grow on the selective screening medium, while co-transformants containing pBX-Rv2031p and pTRG-Rv3133c-deltaC were used as negative controls [19].

### Chromatin immunoprecipitation (ChIP) assays

Actively growing cultures were treated with 1% (v/v) formaldehyde at room temperature for 10 minutes under gentle shaking and the reaction was stopped by adding glycine (at a final concentration of 0.125 M) for 10 minutes with gentle shaking. Cross-linked cells were collected by centrifugation and washed twice with PBS and once with TBSTT (150 mM NaCl, 20 mM Tris-HCl pH 7.5, 10 mM EDTA, 0.1% (v/v) Triton X-100, 0.1% (v/v) Tween 20) to remove excess formaldehyde. Cells were resuspended in 1 ml of TBSTT, sonicated on ice with 20 pulses of 20 seconds and 40% amplitude. The average DNA fragment size obtained was approximately 0.5 kb. Cell debris was removed by centrifugation and the clear supernatant was stored as total lysate. Typically, 100 µl of the supernatant was used as input. 900 µl of the supernatant was incubated for 3 h on a rocker at 4°C with 10 µl of antibodies against OxyS, and then the complexes were immunoprecipitated with 20 µl 50% protein A-agarose for 1 h on a rocker at 4°C. A parallel ChIP experiment without the OxyS antibody was set up as a negative control. The immunocomplex was recovered by centrifugation, washed five times with TBSTT, and resuspended with 100 µl TE buffer (20 mM Tris-HCl pH 7.8, 10 mM EDTA, 0.5% (w/v) SDS). Crosslinking was reversed at 65°C for 6 h. The DNA samples were purified, resuspended in 50 µl TE buffer and analyzed by PCR. Each experiment was performed in duplicate, and repeated twice. The amplified promoter fragments were 350 bp in length. The amplification protocol included one cycle of 5 min at 95°C, and 32 cycles of three steps each: 1 min at 95°C, 1 min at 60°C, and 1 min at 72°C. Primer sequences are indicated in Table S1.

### Electrophoretic mobility shift assays (EMSA)

The binding of OxyS to the *katG* promoter was investigated using a modified electrophoretic mobility shift assay as previously published [30] with the following changes. The forward primers were FITC-labeled at their 5′ termini (Table S1). The double-stranded substrates were prepared according to a previously published procedure [30]. The reactions (10 µl) for measuring mobility shift contained FITC-labeled DNA substrate and various amounts of OxyS at different concentrations diluted in a buffer containing 20 mM Tris-HCl, pH 7.5, 100 mM NaCl, and 2 mM EDTA. Reactions were performed at room temperature for 20 min, and loaded onto 6% polyacrylamide/bis (37.5∶1) gels in 0.5×Tris-borate-EDTA (TBE) buffer, and run at a constant voltage of 130 V for 60 min. Images of the gels were acquired using a Typhoon scanner (GE Healthcare).

### DNase I footprinting assays

The 100 bp (foot1) and 110 bp (foot2) *katG* promoter regions were amplified from genomic DNA using the primers foot1f and foot1r, and foot2f and foot2r, respectively. Both foot1f and foot2f were FITC-labeled (Table S1). The purified substrates were then subjected to the same binding reaction as in the electrophoretic mobility shift assay described above. DNase I footprinting assays were performed as previously described [32]. The ladders for foot1 and foot2 were produced using the Sanger dideoxy method using foot1f and foot2f primers, respectively.

### Quantitative real-time PCR (qRT-PCR)

For real-time PCR analysis, gene-specific primers (Table S1) were used and first-strand cDNAs were synthesized using the SuperScript II reverse transcriptase (Invitrogen) according to the manufacturer's instructions. Each PCR reaction (20 µl) contained 10 µl of 2× SYBR Green Master Mix Reagent (Applied Biosystems), 1.0 µl of cDNA samples, and 200 nM gene-specific primers. The reactions were performed in Bio-Rad IQ5 Real-time PCR machine. The thermocycling conditions were as follows: 95°C for 5 min, and 40 cycles at 95°C for 30 s, 60°C for 30 s and 72°C for 30 s. Amplification specificity was assessed by conducting a melting curve analysis. Gene expression levels were normalized to the levels of 16S rRNA transcripts. The degrees of change in expression were calculated using the 2^−ΔΔCt^ method [33].

### Bacterial growth time course assays

The antimicrobial activity of H_2_O_2_ against *M. smegmatis* was determined using a modified bacterial growth time course assay. *M. smegmatis* was grown in LB at 37°C overnight. This culture was then diluted (1∶100) in 5 ml of fresh LB broth containing the indicated concentration of H_2_O_2_, and the culture was again incubated at 37°C with shaking at 220 rpm for three days. Samples were taken at various time points (0, 12, 24, 36, 48, and 60 h). All assays were performed three times.

## Supporting Information

Table S1 **Primers used in this study.**
(DOC)

Table S2 **Plasmids and recombinant vectors used in this study.**
(DOC)

Figure S1 **Alignment of protein sequences of OxyS and its identified orthologs across the mycobacteria.** Amino acid alignments of orthologous proteins of OxyS from different mycobacteria were performed using local BioEdit software. Residues identical for all proteins are boxed in black, and residues similar for all proteins are boxed in gray. Cysteine residues and the helix-turn-helix motif were indicated. M. tu, *Mycobacterium tuberculosis* H37Rv; M. bo, *Mycobacterium bovis* AF2122/97; M. ka, *Mycobacterium kansasii* ATCC 12478; M. ma, *Mycobacterium marinum* M; M. le, *Mycobacterium leprae* TN; M. av, *Mycobacterium avium* subsp. paratuberculosis K-10; M. in, *Mycobacterium intracellulare* ATCC 13950; M. ul, *Mycobacterium ulcerans* Agy99; M. sm, *Mycobacterium smegmatis* str. mc^2^155.
(TIF)

Figure S2 **Alignment of promoter sequences of** ***katG*** **gene in** ***M. tuberculosis*** **(Rv1908c_up) and** ***M. smegmatis*** **(MSMEG_6384_up).** The conserved OxyS binding site (OxyS box1) was indicated.
(TIF)

Figure S3 **Expression and purification of OxyS and its mutant proteins.** His-tagged OxyS and its mutant proteins were expressed and affinity purified as described under “Materials and Methods”. Proteins were resolved in 12% SDS-PAGE and the gel was stained with coomassie blue. (**A**) Lane 1, uninduced lysate; lane 2 to lane 6, induced lysate. Protein expression was induced at at 20°C for 15 hours after 0.5 mM IPTG (isopropyl β-D-1-thiogalactopyranoside) was added. (**B**) Affinity purified His-OxyS and its mutant proteins. The samples are indicated at the top of the figure. Bands of the correct size are indicated by an arrow on the right of the panel.
(TIF)

Figure S4 **Mapping the binding regions for OxyS in the** ***katG*** **promoter.** (**A**) Schematic representation of several short DNA fragments generated in this study. *katGp*, *katGp1* and *katGp2* were used as DNA substrates in EMSA assays. Both foot1 and foot2 were used as DNA substrates in DNase I footprinting assays. (**B**) EMSA assays for the interactions of OxyS with different DNA fragments. OxyS bound to *katGp* and *katGp2*, but not *katGp1*. The EMSA reactions (10 µl) for measuring mobility shift contained FITC-labeled DNA substrate and increasing amount of OxyS (100 nM, 200 nM, 300 nM and 400 nM). The protein/DNA complexes are indicated by arrows on the right of the panels. (**C**) Unlabeled DNA substrates were used to compete with the FITC-labeled DNA. Unlabeled *katGp2*, but not *katGp1*, could competitively inhibit the binding of OxyS to the FITC-labeled *katGp*. The protein/DNA complexes are indicated by arrows on the right of the panels.
(TIF)

Figure S5 **Interaction of OxyS with** ***M. smegmatis katG*** **promoter (** ***MsmkatGp*** **).** (**A**) ChIP assays for the interaction of OxyS with the *MsmkatG* promoter *in vivo*. DNA recovered from the immunoprecipitates was amplified with primers specific for either *MsmkatGp* or a negative control promoter *MSMEG_1432p*. ‘+’ refers to the immunoprecipitate obtained with OxyS antibodies, whereas ‘−’ refers to the control in which ChIP was carried out without any primary antibodies. ‘Input’ refers to total genomic DNA prior to IP reaction and was used as a positive control in PCR. (**B**) EMSA assays for the interaction of OxyS with *MsmkatGp*. The EMSA reactions (10 µl) for measuring mobility shift contained FITC-labeled DNA substrate and increasing amount of OxyS (100 nM, 200 nM, 300 nM and 400 nM). Unlabeled *MsmkatGp* was used to compete with the FITC-labeled DNA. The protein/DNA complexes are indicated by arrows on the right of the panels.
(TIF)

Figure S6 **Effect of H_2_O_2_ or DTT on the electrophoretic mobilities of OxyS and its mutant proteins was measured by native-PAGE.** Purified *M. tuberculosis* OxyS and its mutants (1.5 µg) were run in the first lane. Equivalent samples were mixed with either DTT (lane 2) or H_2_O_2_ (lane 3) at the concentrations of 3 mM for 30 min at room temperature, respectively. Native PAGE for OxyS and its mutant proteins were performed at room temperature with the use of 12% non-denaturing polyacrylamide gels. The oxidized and reduced protein bands were indicated on the right of the panels.
(TIF)

Figure S7 **Effects of H_2_O_2_ on the growth of recombinant mycobacterial strains measured by detailed bacterial growth time course assays.** Recombinant mycobacterial strains were treated with 0 mM, 1 mM, 2 mM, 3 mM and 4 mM H_2_O_2_. Aliquots were taken at the indicated times. Each analysis was performed in triplicate. Symbols are the average of three replicates, and error bars indicate the SDs (Standard Deviation) of three replicate samples. The recombinant mycobacterial strains are indicated by black boxes (Msm/pMV261) or hollow triangles (Msm/pMV261-OxyS), respectively.
(TIF)

Figure S8 **Assays of cell morphology by scanning electron microscopy.** *M. smegmatis* cells prepared for scanning electron microscopy (SEM) were grown in LB at 37°C for 24 hours. After giving a heat shock at 42°C for 1 hour and incubating at 37°C for additional 4 hours, the cells were harvested by centrifugation. The bacterial pellets were then resuspended and incubated at 4°C for 24 hours in 2.5% (v/v) glutardialdehyde solution. The cells were washed twice in double-distilled water and then dehydrated with a series of 15 min treatments in 30, 50, 75, 85, 95 and 100% ethanol respectively. The final treatment in 100% ethanol was repeated to ensure complete dehydration. Samples were critical-point dried, sputter-coated with gold, and observed using a scanning electron microscope (S570; Hitachi, Tokyo, Japan). The images were taken at 15,000× magnification (bars, 1 µm).
(TIF)

## References

[1] Nguyen L, Pieters J (2005). The Trojan horse: survival tactics of pathogenic mycobacteria in macrophages.. Trends Cell Biol.

[2] Manca C, Paul S, Barry CE, Freedman VH, Kaplan G (1999). *Mycobacterium tuberculosis* catalase and peroxidase activities and resistance to oxidative killing in human monocytes *in vitro*.. Infect Immun.

[3] Yu K, Mitchell C, Xing Y, Magliozzo RS, Bloom BR (1999). Toxicity of nitrogen oxides and related oxidants on mycobacteria: *M. tuberculosis* is resistant to peroxynitrite anion.. Tuber Lung Dis.

[4] Aderem A, Underhill DM (1999). Mechanisms of phagocytosis in macrophages.. Annu Rev Immunol.

[5] Jittawuttipoka T, Buranajitpakorn S, Vattanaviboon P, Mongkolsuk S (2009). The catalase-peroxidase KatG is required for virulence of *Xanthomonas campestris* pv. campestris in a host plant by providing protection against low levels of H_2_O_2_.. J Bacteriol.

[6] Zhang Y, Heym B, Allen B, Young D, Cole S (1992). The catalase-peroxidase gene and isoniazid resistance of *Mycobacterium tuberculosis*.. Nature.

[7] Pym AS, Domenech P, Honoré N, Song J, Deretic V (2001). Regulation of catalase-peroxidase (KatG) expression, isoniazid sensitivity and virulence by *furA* of *Mycobacterium tuberculosis*.. Mol Microbiol.

[8] Milano A, Forti F, Sala C, Riccardi G, Ghisotti D (2001). Transcriptional regulation of *furA* and *katG* upon oxidative stress in *Mycobacterium smegmatis*.. J Bacteriol.

[9] Sala C, Forti F, Di Florio E, Canneva F, Milano A (2003). *Mycobacterium tuberculosis* FurA autoregulates its own expression.. J Bacteriol.

[10] Sala C, Forti F, Magnoni F, Ghisotti D (2008). The *katG* mRNA of *Mycobacterium tuberculosis* and *Mycobacterium smegmatis* is processed at its 5′ end and is stabilized by both a polypurine sequence and translation initiation.. BMC Mol Biol.

[11] Zahrt TC, Song J, Siple J, Deretic V (2001). Mycobacterial FurA is a negative regulator of catalase-peroxidase gene *katG*.. Mol Microbiol.

[12] Master S, Zahrt TC, Song J, Deretic V (2001). Mapping of *Mycobacterium tuberculosis katG* promoters and their differential expression in infected macrophages.. J Bacteriol.

[13] Lee HI, Yoon JH, Nam JS, Kim YM, Ro YT (2010). Cloning, expression and characterization of the catalase-peroxidase (KatG) gene from a fast-growing *Mycobacterium sp.* strain JC1 DSM 3803.. J Biochem.

[14] Italiani VC, da Silva Neto JF, Braz VS, Marques MV (2011). Regulation of catalase-peroxidase KatG is OxyR dependent and Fur independent in *Caulobacter crescentus*.. J Bacteriol.

[15] Pagán-Ramos E, Song J, McFalone M, Mudd MH, Deretic V (1998). Oxidative stress response and characterization of the *oxyR-ahpC* and *furA-katG* loci in *Mycobacterium marinum*.. J Bacteriol.

[16] Kim SO, Merchant K, Nudelman R, Beyer WF, Keng T (2002). OxyR: a molecular code for redox-related signaling.. Cell.

[17] Domenech P, Honoré N, Heym B, Cole ST (2001). Role of OxyS of *Mycobacterium tuberculosis* in oxidative stress: overexpression confers increased sensitivity to organic hydroperoxides.. Microbes Infect.

[18] Maddocks SE, Oyston PC (2008). Structure and function of the LysR-type transcriptional regulator (LTTR) family proteins.. Microbiology.

[19] Guo M, Feng H, Zhang J, Wang W, Wang Y (2009). Dissecting transcription regulatory pathways through a new bacterial one-hybrid reporter system.. Genome Res.

[20] Chen H, Xu G, Zhao Y, Tian B, Lu H (2008). A novel OxyR sensor and regulator of hydrogen peroxide stress with one cysteine residue in *Deinococcus radiodurans*.. PLoS One.

[21] Green J, Paget MS (2004). Bacterial redox sensors.. Nat Rev Microbiol.

[22] Singh A, Guidry L, Narasimhulu KV, Mai D, Trombley J (2007). *Mycobacterium tuberculosis* WhiB3 responds to O_2_ and nitric oxide via its (4Fe-4S) cluster and is essential for nutrient starvation survival.. Proc Natl Acad Sci USA.

[23] Zheng M, Aslund F, Storz G (1998). Activation of the OxyR transcription factor by reversible disulfide bond formation.. Science.

[24] Christman MF, Storz G, Ames BN (1989). OxyR, a positive regulator of hydrogen peroxide-inducible genes in *Escherichia coli* and *Salmonella typhimurium*, is homologous to a family of bacterial regulatory proteins.. Proc Natl Acad Sci USA.

[25] Deretic V, Philipp W, Dhandayuthapani S, Mudd MH, Curcic R (1995). *Mycobacterium tuberculosis* is a natural mutant with an inactivated oxidative-stress regulatory gene: implications for sensitivity to isoniazid.. Mol Microbiol.

[26] Sherman DR, Sabo PJ, Hickey MJ, Arain TM, Mahairas GG (1995). Disparate responses to oxidative stress in saprophytic and pathogenic mycobacteria.. Proc Natl Acad Sci USA.

[27] Sreevatsan S, Escalante P, Pan X, Gillies DA, Siddiqui S (1996). Identification of a polymorphic nucleotide in *oxyR* specific for *Mycobacterium bovis*.. J Clin Microbiol.

[28] Dhandayuthapani S, Zhang Y, Mudd MH, Deretic V (1996). Oxidative stress response and its role in sensitivity to isoniazid in mycobacteria: characterization and inducibility of *ahpC* by peroxides in *Mycobacterium smegmatis* and lack of expression in *M. aurum* and *M. tuberculosis*.. J Bacteriol.

[29] Zhang H, Deng JY, Bi LJ, Zhou YF, Zhang ZP (2008). Characterization of *Mycobacterium tuberculosis* nicotinamidase/pyrazinamidase.. FEBS J.

[30] He ZG, Rezende LF, Willcox S, Griffith JD, Richardson CC (2003). The carboxyl-terminal domain of bacteriophage T7 single-stranded DNA-binding protein modulates DNA binding and interaction with T7 DNA polymerase.. J Biol Chem.

[31] Gill SC, Hippel PH (1989). Calculation of protein extinction coefficients from amino acid sequence data.. Anal Biochem.

[32] Li Y, Zeng J, Zhang H, He ZG (2010). The characterization of conserved binding motifs and potential target genes for *M. tuberculosis* MtrAB reveals a link between the two-component system and the drug resistance of *M. smegmatis*.. BMC Microbiol.

[33] Livak KJ, Schmittgen TD (2001). Analysis of relative gene expression data using real-time quantitative PCR and the 2(−Delta Delta C(T)) Method.. Methods.

[34] Arnold K, Bordoli L, Kopp J, Schwede T (2006). The SWISS-MODEL workspace: a web-based environment for protein structure homology modelling.. Bioinformatics.

[35] Muraoka S, Okumura R, Ogawa N, Nonaka T, Miyashita K (2003). Crystal structure of a full-length LysR-type transcriptional regulator, CbnR: unusual combination of two subunit forms and molecular bases for causing and changing DNA bend.. J Mol Biol.

[36] Stover CK, de la Cruz VF, Fuerst TR, Burlein JE, Benson LA (1991). New use of BCG for recombinant vaccines.. Nature.

