# Supplementary material for: The Mycobacterial LysR-Type Regulator OxyS Responds to Oxidative Stress and Negatively Regulates Expression of the Catalase-Peroxidase Gene
Source: PLoS One. 2012 Jan 17;7(1):e30186. doi: 10.1371/journal.pone.0030186 (PMC3260234; doi:10.1371/journal.pone.0030186)
Supplement: Table S1 — Primers used in this study. (DOC) [file pone.0030186.s001.doc]

**Table S**1. Primers used in this study.

| **Used in** | **Construct** | **Primer name** | **Sequence (from 5' to 3')** |
| --- | --- | --- | --- |
| Cloning and expression | OxyS | *R0117-f*  *R0117-r* | ATAAGCGGCCGCAGTGCTCTTCCGTCAGCTGGA  CAGATCTAGAGAGTCAGCGGCGACGGGTGATCC |
| site-directed mutagenesis | OxyS-C25A | *C25A-f*  *C25A-r* | GCCGCTGAGAAGGCCTACGTGTCGCAA  TTGCGACACGTAGGCCTTCTCAGCGGC |
| site-directed mutagenesis | OxyS-C113A | *C113A-f*  *C113A-r* | CTGTCGGCGTTTGCCTCGGCGCACCCG  CGGGTGCGCCGAGGCAAACGCCGACAG |
| site-directed mutagenesis | OxyS-C124A | *C124A-f*  *C124A-r* | AAGGTGCAAGTCGCTTCCCGGCTGGCT  AGCCAGCCGGGAAGCGACTTGCACCTT |
| site-directed mutagenesis | OxyS-C293A | *C293A-f*  *C293A-r* | GCGCTCATAACAGCCGCGCAGGCGCTG  CAGCGCCTGCGCGGCTGTTATGAGCGC |
| ChIP | katGp | *Rv1908cpf*  *Rv1908cpr* | ATATGAATTCGCGTTTTGCGCTGCCCGACG  GCGCTCTAGAAGCATTCCTTCCAGGAGTTG |
| ChIP | Rv3911p | *Rv3911pf*  *Rv3911pr* | GCGCGAATTCGTTCCCTAGCTTCAAGAACG  AGATTCTAGAAGCACTTCACCCTGGCTGGC |
| ChIP | MsmkatGp | *msmeg_6384pf*  *msmeg_6384pr* | ATATGAATTCTCGTGACCCACACGACCGAC  GACGTCTAGATGCATTTCCTTTCGGGAGTG |
| ChIP | MSMEG_1432p | *msmeg_1432pf*  *msmeg_1432pr* | AGATGAATTCCTTCGGGCGGAACCAGTCGG  ATATTCTAGAGCGCTGAGCCTACTGGGCCG |
| EMSA | FITC-katGp | *katGpf*  *katGpr* | FITC-GCGTTTTGCGCTGCCCGACGTATCC  AGCATTCCTTCCAGGAGTTGGTGTT |
| EMSA | FITC-katGp1 | *katGpf*  *katGp1r* | FITC-GCGTTTTGCGCTGCCCGACGTATCC  TCGACATCGGCGATAACCCCGCAAG |
| EMSA | FITC-katGp2 | *katGp2f*  *katGpr* | FITC-CCGATGTCGACTGTGCTGTTGGCGA  AGCATTCCTTCCAGGAGTTGGTGTT |
| EMSA | FITC-OxySbox1 | *box1f*  *box1r* | FITC-TGTCTGACGGCCTCGGACCAT  ATGGTCCGAGGCCGTCAGACA |
| EMSA | FITC-  OxySbox1mut | *box1mutf*  *box1mutr* | FITC-TGTCCCACGGCCTCGCCCCAT  ATGGGGCGAGGCCGTGGGACA |
| DNase I footprinting | foot1 | *foot1f*  *foot1r* | FITC-TATACGGAATTCCCGATGTCGACTGTGCTGTT  CATAGACCCCAGTAGATGAC |
| DNase I footprinting | foot2 | *foot2f*  *foot2r* | FITC-TATACGGAATTCCGAGGCGGAGGTCATCTACT  AGCATTCCTTCCAGGAGTTG |
| qRT-PCR | MSMEG_3757 | *msmeg_3757RTf*  *msmeg_3757RTr* | AGCAGCCGCGGTAATACG  CGAGCTCTTTACGCCCAGTAA |
| qRT-PCR | MSMEG_6383 | *msmeg_6383RTf*  *msmeg_6383RTr* | TGACCCACACGACCGACTT  CGTACACGGACCGCAACAC |
| qRT-PCR | MSMEG_6384 | *msmeg_6384RTf*  *msmeg_6384RTr* | CCCAATCAGCTCAATCTGAAGA  CTCGTCCAGCGGATTGATG |
