# Supplementary material for: The Mycobacterial LysR-Type Regulator OxyS Responds to Oxidative Stress and Negatively Regulates Expression of the Catalase-Peroxidase Gene
Source: PLoS One. 2012 Jan 17;7(1):e30186. doi: 10.1371/journal.pone.0030186 (PMC3260234; doi:10.1371/journal.pone.0030186)
Supplement: Table S2 — Plasmids and recombinant vectors used in this study. (DOC) [file pone.0030186.s002.doc]

**Table S2. Plasmids and recombinant vectors used in this study.**

| **Plasmids** | **Description** | **Sources** |
| --- | --- | --- |
| pBT | Bacterial two-hybird assay bait domain vector | Stratagene |
| pTRG | Bacterial two-hybird assay target domain vector | Stratagene |
| pTRG-OxyS | pTRG derivative for Bacterial one-hybird assay | This work |
| pET28a | Kan^r^ expression vector with 6His-tag coding sequence | Novagen |
| pET-OxyS | pET derivative for expression 6His-OxyS | This work |
| pET-OxyS-C25A | pET derivative for expression 6His-OxyS-C25A | This work |
| pET-OxyS-C113A | pET derivative for expression 6His-OxyS-C113A | This work |
| pET-OxyS-C124A | pET derivative for expression 6His-OxyS-C124A | This work |
| pET-OxyS-C293A | pET derivative for expression 6His-OxyS-C293A | This work |
| pBXcmT | pBT derived plasmid for detection of protein-DNA interaction | *Guo et al*, 2009 |
| pBX-*katGp* | pBXcmT derivative for Bacterial one-hybird assay | This work |
| pBX-*Rv3911cp* | pBXcmT derivative for Bacterial one-hybird assay | This work |
| pMV261 | A inducible system for conditional gene over-expression in mycobacteria | *Stover et al, 1991* |
| pMV261-OxyS | pMV261 derivative for over-expression of *oxyS* in *M.sm* | This work |
