# Supplementary figures and images for: The Mycobacterial LysR-Type Regulator OxyS Responds to Oxidative Stress and Negatively Regulates Expression of the Catalase-Peroxidase Gene
Source: PLoS One. 2012 Jan 17;7(1):e30186. doi: 10.1371/journal.pone.0030186 (PMC3260234; doi:10.1371/journal.pone.0030186)

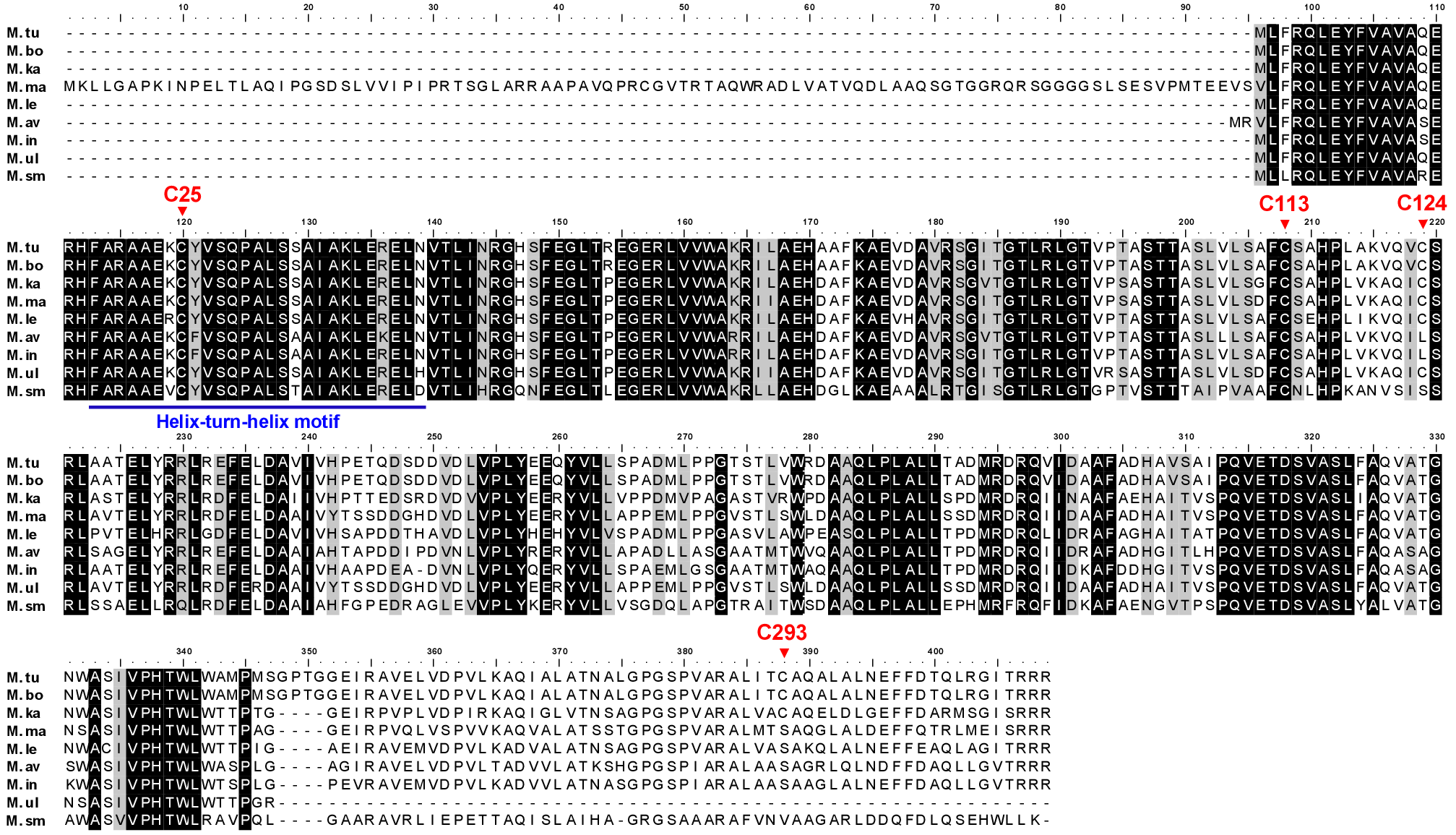

Supplement: Figure S1 — Alignment of protein sequences of OxyS and its identified orthologs across the mycobacteria. Amino acid alignments of orthologous proteins of OxyS from different mycobacteria were performed using local BioEdit software. Residues identical for all proteins are boxed in black, and residues similar for all proteins are boxed in gray. Cysteine residues and the helix-turn-helix motif were indicated. M. tu, Mycobacterium tuberculosis H37Rv; M. bo, Mycobacterium bovis AF2122/97; M. ka, Mycobacterium kansasii ATCC 12478; M. ma, Mycobacterium marinum M; M. le, Mycobacterium leprae TN; M. av, Mycobacterium avium subsp. paratuberculosis K-10; M. in, Mycobacterium intracellulare ATCC 13950; M. ul, Mycobacterium ulcerans Agy99; M. sm, Mycobacterium smegmatis str. mc2155. (TIF) [file pone.0030186.s003.tif]

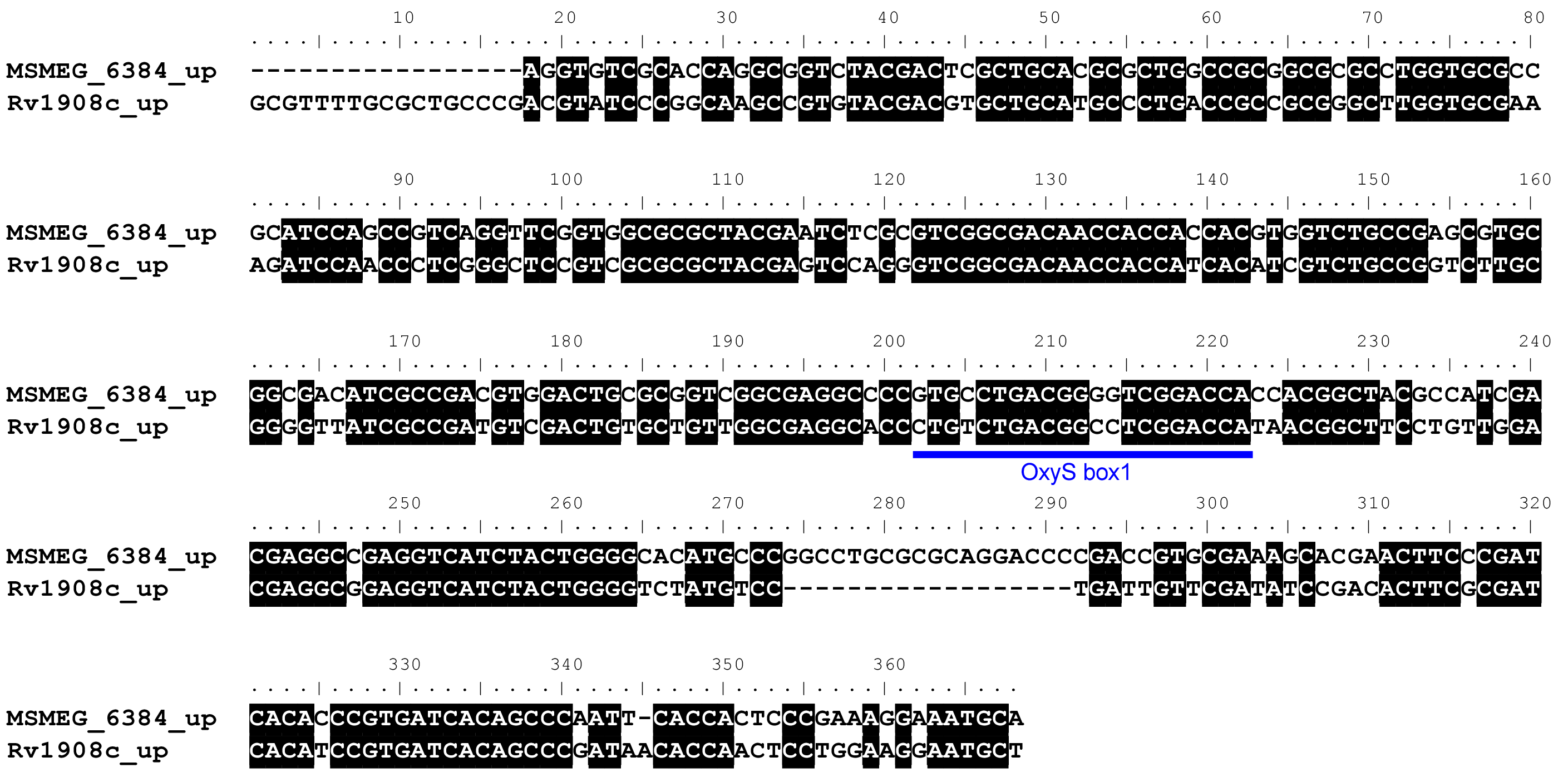

Supplement: Figure S2 — Alignment of promoter sequences of katG gene in M. tuberculosis (Rv1908c_up) and M. smegmatis (MSMEG_6384_up). The conserved OxyS binding site (OxyS box1) was indicated. (TIF) [file pone.0030186.s004.tif]

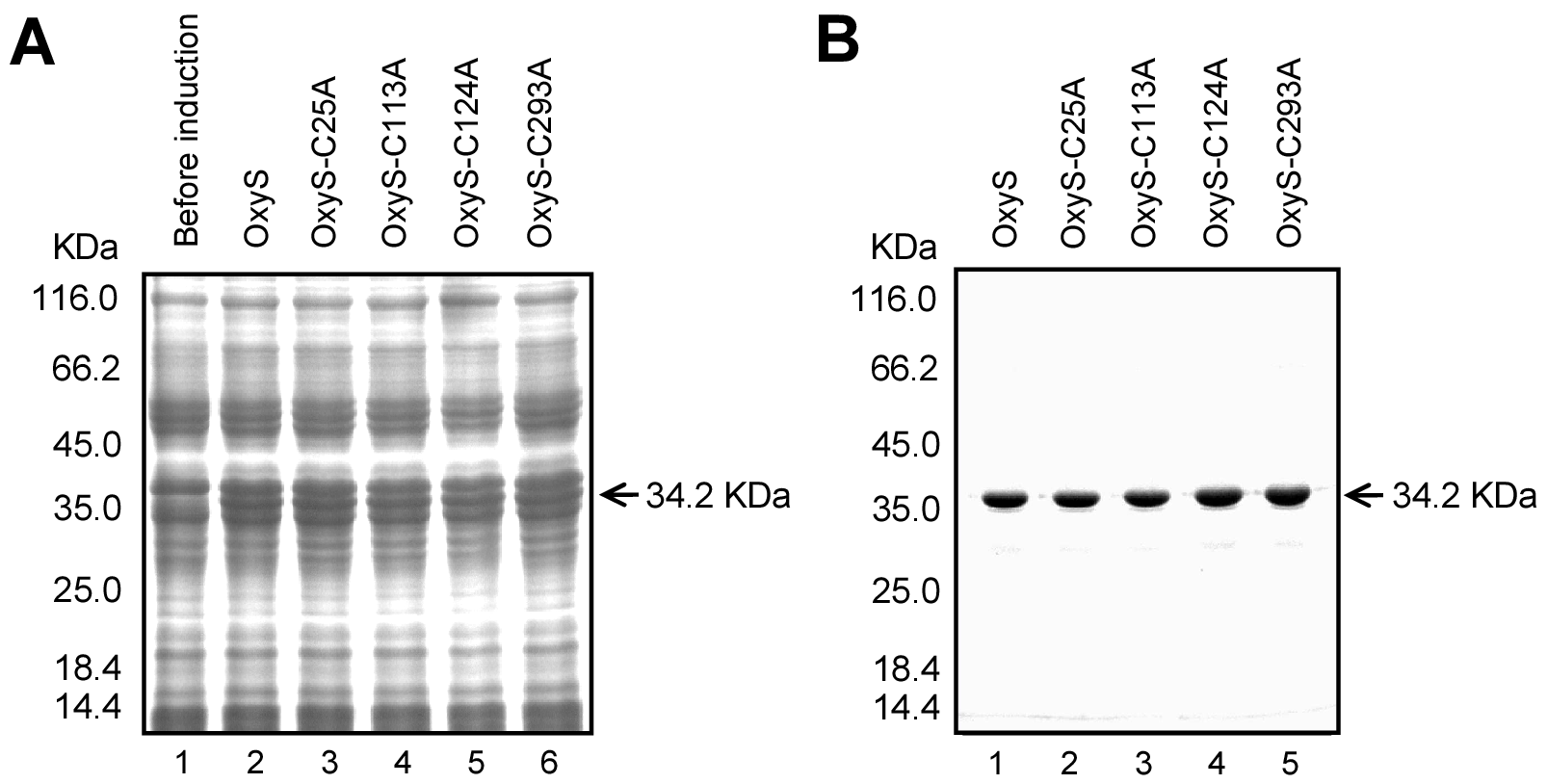

Supplement: Figure S3 — Expression and purification of OxyS and its mutant proteins. His-tagged OxyS and its mutant proteins were expressed and affinity purified as described under “Materials and Methods”. Proteins were resolved in 12% SDS-PAGE and the gel was stained with coomassie blue. (A) Lane 1, uninduced lysate; lane 2 to lane 6, induced lysate. Protein expression was induced at at 20°C for 15 hours after 0.5 mM IPTG (isopropyl β-D-1-thiogalactopyranoside) was added. (B) Affinity purified His-OxyS and its mutant proteins. The samples are indicated at the top of the figure. Bands of the correct size are indicated by an arrow on the right of the panel. (TIF) [file pone.0030186.s005.tif]

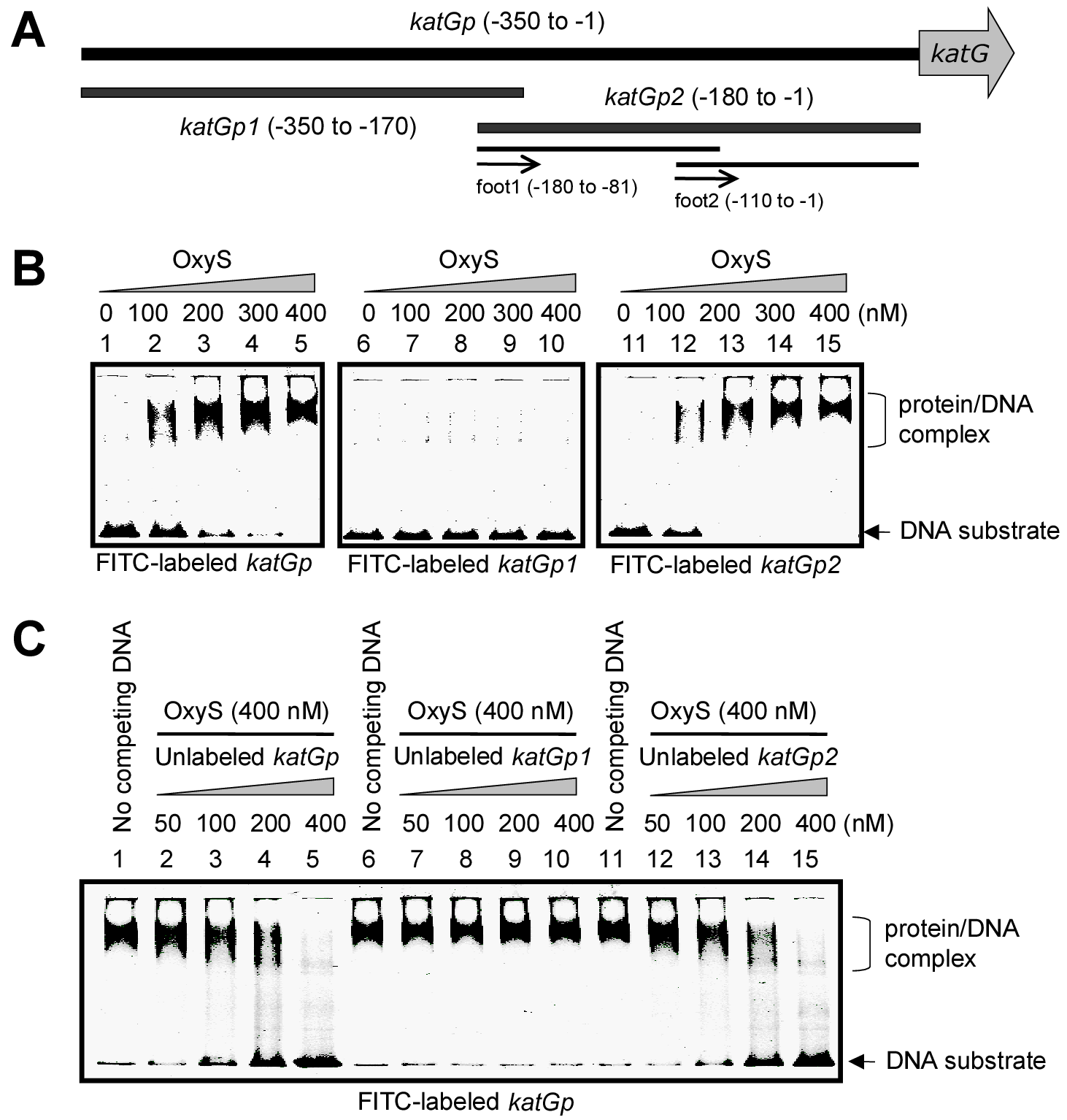

Supplement: Figure S4 — Mapping the binding regions for OxyS in the katG promoter. (A) Schematic representation of several short DNA fragments generated in this study. katGp, katGp1 and katGp2 were used as DNA substrates in EMSA assays. Both foot1 and foot2 were used as DNA substrates in DNase I footprinting assays. (B) EMSA assays for the interactions of OxyS with different DNA fragments. OxyS bound to katGp and katGp2, but not katGp1. The EMSA reactions (10 µl) for measuring mobility shift contained FITC-labeled DNA substrate and increasing amount of OxyS (100 nM, 200 nM, 300 nM and 400 nM). The protein/DNA complexes are indicated by arrows on the right of the panels. (C) Unlabeled DNA substrates were used to compete with the FITC-labeled DNA. Unlabeled katGp2, but not katGp1, could competitively inhibit the binding of OxyS to the FITC-labeled katGp. The protein/DNA complexes are indicated by arrows on the right of the panels. (TIF) [file pone.0030186.s006.tif]

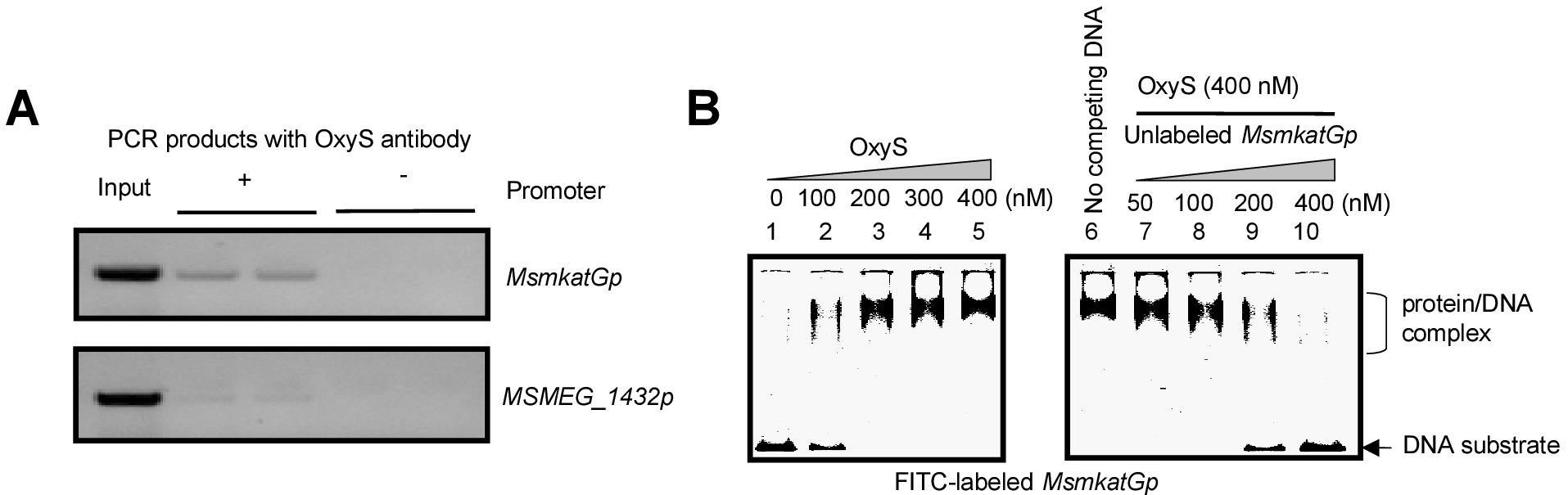

Supplement: Figure S5 — Interaction of OxyS with M. smegmatis katG promoter ( MsmkatGp ). (A) ChIP assays for the interaction of OxyS with the MsmkatG promoter in vivo. DNA recovered from the immunoprecipitates was amplified with primers specific for either MsmkatGp or a negative control promoter MSMEG_1432p. ‘+’ refers to the immunoprecipitate obtained with OxyS antibodies, whereas ‘−’ refers to the control in which ChIP was carried out without any primary antibodies. ‘Input’ refers to total genomic DNA prior to IP reaction and was used as a positive control in PCR. (B) EMSA assays for the interaction of OxyS with MsmkatGp. The EMSA reactions (10 µl) for measuring mobility shift contained FITC-labeled DNA substrate and increasing amount of OxyS (100 nM, 200 nM, 300 nM and 400 nM). Unlabeled MsmkatGp was used to compete with the FITC-labeled DNA. The protein/DNA complexes are indicated by arrows on the right of the panels. (TIF) [file pone.0030186.s007.tif]

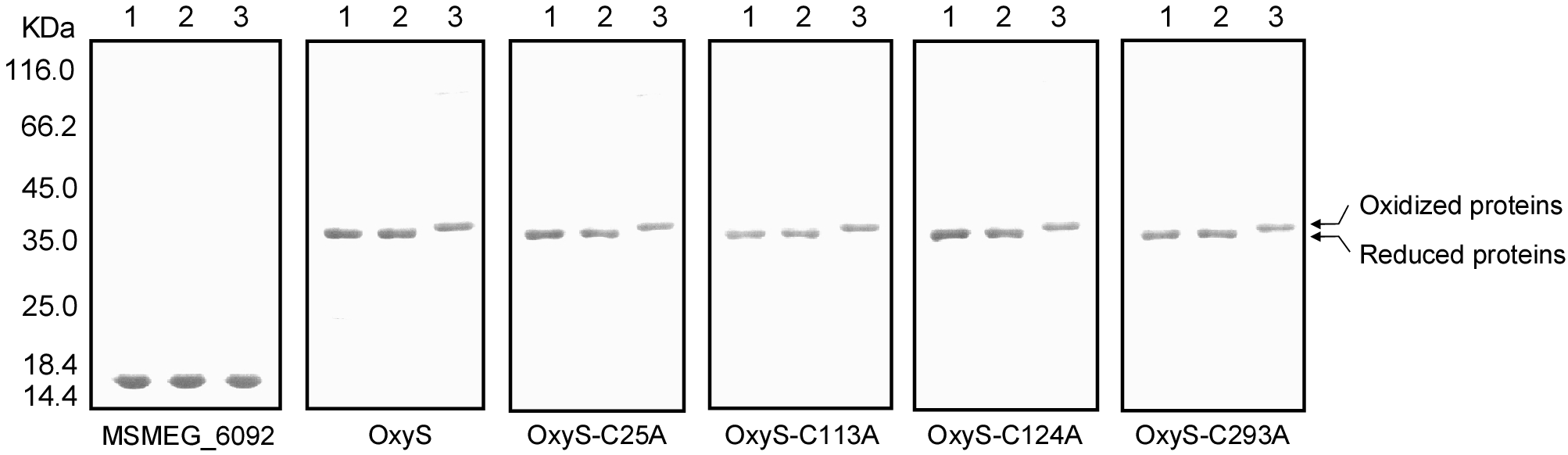

Supplement: Figure S6 — Effect of H2O2 or DTT on the electrophoretic mobilities of OxyS and its mutant proteins was measured by native-PAGE. Purified M. tuberculosis OxyS and its mutants (1.5 µg) were run in the first lane. Equivalent samples were mixed with either DTT (lane 2) or H2O2 (lane 3) at the concentrations of 3 mM for 30 min at room temperature, respectively. Native PAGE for OxyS and its mutant proteins were performed at room temperature with the use of 12% non-denaturing polyacrylamide gels. The oxidized and reduced protein bands were indicated on the right of the panels. (TIF) [file pone.0030186.s008.tif]

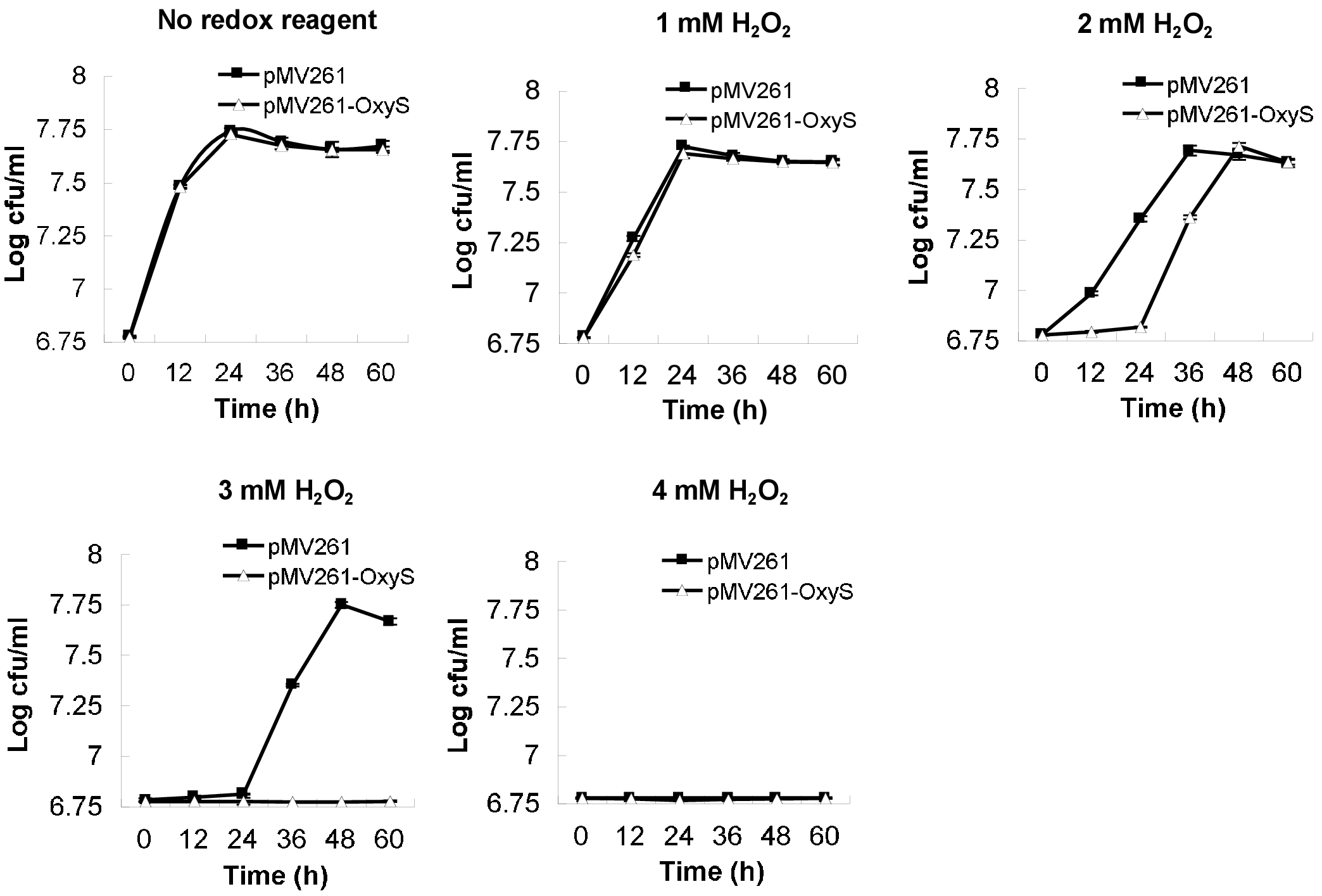

Supplement: Figure S7 — Effects of H2O2 on the growth of recombinant mycobacterial strains measured by detailed bacterial growth time course assays. Recombinant mycobacterial strains were treated with 0 mM, 1 mM, 2 mM, 3 mM and 4 mM H2O2. Aliquots were taken at the indicated times. Each analysis was performed in triplicate. Symbols are the average of three replicates, and error bars indicate the SDs (Standard Deviation) of three replicate samples. The recombinant mycobacterial strains are indicated by black boxes (Msm/pMV261) or hollow triangles (Msm/pMV261-OxyS), respectively. (TIF) [file pone.0030186.s009.tif]

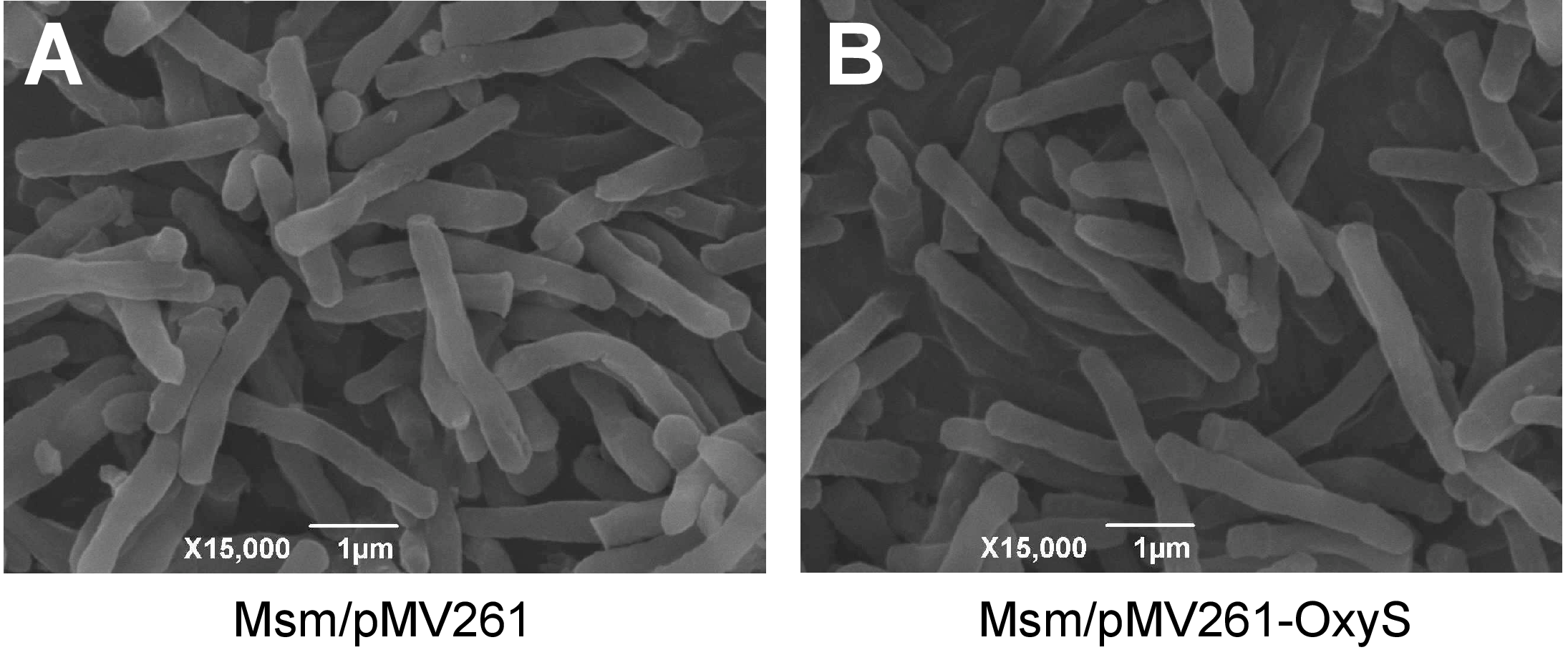

Supplement: Figure S8 — Assays of cell morphology by scanning electron microscopy. M. smegmatis cells prepared for scanning electron microscopy (SEM) were grown in LB at 37°C for 24 hours. After giving a heat shock at 42°C for 1 hour and incubating at 37°C for additional 4 hours, the cells were harvested by centrifugation. The bacterial pellets were then resuspended and incubated at 4°C for 24 hours in 2.5% (v/v) glutardialdehyde solution. The cells were washed twice in double-distilled water and then dehydrated with a series of 15 min treatments in 30, 50, 75, 85, 95 and 100% ethanol respectively. The final treatment in 100% ethanol was repeated to ensure complete dehydration. Samples were critical-point dried, sputter-coated with gold, and observed using a scanning electron microscope (S570; Hitachi, Tokyo, Japan). The images were taken at 15,000× magnification (bars, 1 µm). (TIF) [file pone.0030186.s010.tif]
